# Comparative Effectiveness of Combination Versus Single-Modality Physiotherapy for Rotator Cuff-Related Shoulder Pain: A Systematic Review and Network Meta-Analysis

**DOI:** 10.3390/jcm14134765

**Published:** 2025-07-05

**Authors:** Chien-Sheng Lo, Kuan-Chung Chen, Jui-Chi Shih, Bill Cheng, Wei-Cheng Chao

**Affiliations:** 1 Department of Orthopedics, Chung Shan Medical University Hospital, Taichung City 402, Taiwan; johnlcs317@gmail.com (C.-S.L.); a14651@show.org.tw (K.-C.C.); 2 Department of Orthopedics, Show Chwan Memorial Hospital, Changhua City 500, Taiwan; 3 School of Allied Health, Australian Catholic University, Brisbane, QLD 4014, Australia; s00382872@myacu.edu.au; 4 Graduate Institute of Biomedical Engineering, National Chung Hsing University, Taichung City 402, Taiwan; bcheng@dragon.nchu.edu.tw; 5 Doctoral Program in Tissue Engineering and Regenerative Medicine, College of Medicine, National Chung Hsing University, Taichung City 402, Taiwan

**Keywords:** rotator cuff-related shoulder pain, RCRSP, combination therapy, physiotherapy, pain reduction, functional improvement

## Abstract

**Background/Objective:** The objective of this study is to compare the relative effectiveness of combination therapy (exercise plus manual therapy) versus single-modality physiotherapy interventions for improving pain and function in patients with rotator cuff-related shoulder pain (RCRSP), using a network meta-analysis (NMA) approach. **Methods:** We systematically searched five electronic databases from inception to October 2023 for randomized controlled trials (RCTs) evaluating non-invasive physiotherapy interventions in adults with RCRSP. Primary outcomes included pain intensity and shoulder function, assessed at 12 weeks. A frequentist NMA was conducted to estimate standardized mean differences (SMDs) with 95% confidence intervals (CIs). Risk of bias was assessed using the Cochrane RoB 2.0 tool. **Results:** Eleven RCTs (*n* = 548) were included. Combination therapy demonstrated the greatest improvement in function (SMD = −1.02; 95% CI: −2.59 to 0.56) and pain (SMD = −1.05; 95% CI: −2.41 to 0.30), although the wide confidence intervals crossing the null suggests statistical uncertainty. Exercise therapy alone showed moderate functional improvement (SMD = −0.41; 95% CI: −1.64 to 0.82), and Kinesio taping (KT) provided moderate pain relief (SMD = −0.53; 95% CI: −1.81 to 0.75). While these effects approached known minimal clinically important difference (MCID) thresholds (e.g., DASH: 10–15; VAS: 1.4–2.0), they did not reach statistical significance. **Conclusions:** Based on 11 RCTs, combination therapy (exercise plus manual therapy) appears to be the most effective non-invasive approach for improving pain and function in patients with RCRSP. However, the wide confidence intervals highlight uncertainty. Further large-scale and long-term trials are warranted to confirm its clinical utility and sustainability.

## 1. Introduction

Rotator cuff-related shoulder pain (RCRSP) is one of the most common musculoskeletal disorders worldwide, affecting 10% to 20% of adults, with prevalence rising to as high as 70% in individuals over the age of 50 [1,2]. The condition includes subacromial impingement syndrome (SIS), tendinopathy, and bursitis, typically leading to chronic pain, restricted shoulder mobility, and functional impairment, which in turn affect patients’ daily activities and work productivity [3,4]. Epidemiological studies show that approximately 70% of patients with shoulder pain are diagnosed with RCRSP, and 30% to 40% of these patients continue to experience persistent pain and functional impairment despite receiving treatment [5,6]. Due to its high recurrence rate and long-term impact, RCRSP places a significant burden on the healthcare system. It also leads to reduced workplace productivity and increased costs for businesses and insurance systems. As a result, identifying the most efficient and affordable non-invasive treatment strategies has become a critical need in both clinical practice and the healthcare systems [7,8].

Currently, physiotherapy is the primary non-invasive treatment for RCRSP. Common therapeutic approaches include exercise therapy [9,10], manual therapy [3,11,12], Kinesio taping (KT) [13,14], and combination therapy [6,15]. However, the effectiveness of different physiotherapy approaches remains a topic of debate. While exercise therapy is known to provide the best long-term outcomes, some studies suggest that manual therapy may be more effective in the short term [16,17,18]. In addition, KT may provide short-term pain relief in some patients, but its long-term efficacy remains unclear [2,19]. Whether combination therapy (e.g., exercise therapy plus manual therapy) was superior to single-modality treatment across all clinical outcomes is not supported by comprehensive comparative evidence. The lack of head-to-head comparisons among all available interventions poses challenges for clinicians when determining the most effective treatment option [6,20].

Traditional meta-analyses are limited to comparing two treatments at a time and are unable to simultaneously assess the relative effectiveness of multiple therapies. In contrast, network meta-analysis (NMA) offers a more robust and integrated approach by synthesizing both direct and indirect evidence across a range of treatments. This methodology allows for a comparative ranking of multiple physiotherapy interventions [21]—even in the absence of comprehensive pairwise trials—thereby facilitating more informed clinical decisions [22].

The aim of this study was to conduct a systematic review and network meta-analysis, following PRISMA-NMA guidelines, to compare the relative effectiveness of physiotherapy interventions on pain relief (measured via VAS, NPRS) and functional improvement (SPADI, DASH, Constant Score) in adults with RCRSP. Specifically, the study investigates whether combination therapy (exercise plus manual therapy) was more effective than single-treatment modalities over a follow-up period of at least 12 weeks [23]. The central hypothesis is that combination therapy yields superior functional and pain outcomes compared to either exercise or manual therapy alone. The findings aim to inform evidence-based clinical recommendations and guide optimal non-surgical management strategies to improve outcomes for patients and reduce the long-term burden of RCRSP.

## 2. Materials and Methods

### 2.1. Database Searches and Study Identification

This study was conducted following the Preferred Reporting Items for Systematic Reviews and Meta-Analyses (PRISMA) extension guidelines for network meta-analysis (PRISMA NMA) [24]. No ethical committee approval or informed consent from participants was required. Two authors conducted electronic searches in PubMed, Embase, Cochrane Reviews, Cochrane CENTRAL, and ClinicalTrials.gov databases separately using the following keywords: (‘Rotator cuff related shoulder pain’ or ‘RCRSP’) AND (‘subacromial impingement syndrome’ or ‘SIS’) AND (‘shoulder impingement syndrome ‘ or ‘SIS’) AND (‘physical therapy‘ or ‘rehabilitation‘) AND (‘Pain’ OR ‘Vas’) AND (‘DASH ‘ OR ‘Disabilities of the Arm, Shoulder, and Hand‘). Searches for the systematic reviews and network meta-analyses spanned from database inception to 7 April 2025.

During the initial screening phase, two authors independently assessed the titles and abstracts of identified articles and resolved discrepancies through consensus. Eligible trials were identified through searches conducted in the databases listed above. In addition, the cited references within selected review papers [1,4,5,19,25,26] were assessed, and manual searches were conducted. A third author was consulted to resolve any unresolved disagreements. No limitations were placed on publication language during the searches.

### 2.2. Inclusion and Exclusion Criteria

The PICO model (population, intervention, comparison, outcome) guided the design of this network meta-analysis with the criteria as follows: (1) P: patients diagnosed with rotator cuff-related shoulder pain (RCRSP); (2) I: physiotherapy modalities (e.g., exercise therapy, manual therapy, modality therapy, combination therapy: exercise plus manual); (3) C: other physiotherapy modalities or standard treatment (e.g., placebo, control group); (4) O: primary outcomes: pain reduction (VAS, NPRS), secondary outcomes: functional improvement (SPADI, DASH, Constant Score).

**Figure 1:**
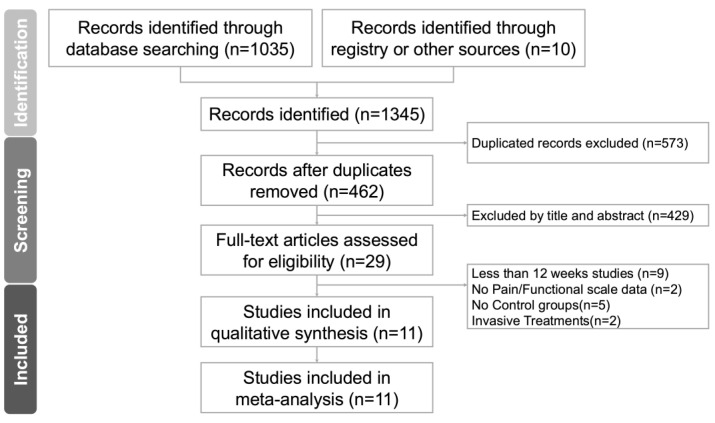
The PRISMA flow diagram of the study selection process.

The inclusion criteria primarily focused on the following aspects: To be included in this review, studies must meet the following criteria: (1) they must be randomized controlled trials (RCTs); (2) the study population must consist of adults diagnosed with RCRSP; (3) the study must include at least one physiotherapy intervention, compared to another physiotherapy intervention or standard treatment; (4) the study must provide quantifiable data, including pain and functional assessment outcomes; (5) the study must last for at least 12 weeks, including treatment and follow-up (Figure 1_PRISMA).

Choice of a 12-week evaluation period was informed by an initial review of the literature, which identified it as the most frequently reported follow-up duration among the included studies. Additionally, previous large-scale analyses have shown that the effects of exercise-based rehabilitation typically emerge around 12 weeks [26]. To enable consistent comparisons across interventions, a standardized assessment time point was necessary. Therefore, this study focuses on the 12-week timeframe and excludes durations with limited available data [23].

Interventions assessed were as follows: This study includes the following physiotherapy modalities: exercise therapy: shoulder stabilization training, strengthening exercises; manual therapy: joint mobilization, massage therapy; physical modalities: electrical stimulation (TENS, EMS), shock wave therapy, and KT; combination therapy: a combination of different physiotherapy modalities (e.g., exercise plus manual therapy) [27].

Population: The targeted population included individuals aged 18 to 75 years with RCRSP, excluding acute trauma, complete rotator cuff tears, and post-surgical patients.

Study design: The included studies needed to have quantitative measures and detailed reports on outcomes such as pain reduction and functional improvement.

The exclusion criteria for this study included the following:

Study duration: studies with a treatment or follow-up period of less than 12 weeks.

Outcome measures: studies that do not report pain or functional scale data (e.g., VAS, NPRS, DASH).

Control groups: studies without a control group or a comparison intervention.

Invasive treatments: studies involving surgical interventions, pharmacological injections (e.g., corticosteroid injections), or other invasive procedures.

These exclusion criteria were chosen to ensure the reliability and relevance of the study. A minimum duration of 12 weeks ensures sufficient time for the effects of physiotherapy to manifest. Studies lacking pain or functional scale data were excluded due to the absence of objective outcome measures for comparison. The absence of control groups also prevented valid comparisons between interventions. Lastly, invasive treatments (e.g., surgery, injections) were excluded to focus solely on the effectiveness of non-invasive physiotherapy.

### 2.3. Standardization for Comparison

Standardization ensures consistency across studies by normalizing pain (VAS/NPRS) and function (SPADI/DASH/Constant Score) scores to a 0–100 scale. Only 12-week outcome data will be used for comparison. Physiotherapy interventions will be classified into distinct categories (e.g., exercise, manual therapy, electrotherapy). Effect sizes will be reported as mean difference (MD) or standardized mean difference (SMD), with 95% confidence intervals (CIs). Comparisons will be made against placebo, standard care, or alternative therapies.

### 2.4. Modeling for Network Meta-Analysis

The transitivity assumption was considered plausible because the included studies were sufficiently similar in terms of key clinical and methodological features. All studies recruited adult participants diagnosed with rotator cuff-related shoulder pain (RCRSP), applied non-invasive physiotherapy interventions, and assessed outcomes using validated scales for pain (VAS or NPRS) and function (DASH or SPADI) within a standardized 12-week timeframe.

Potential effect modifiers—such as participant age, symptom chronicity, baseline shoulder function, and intervention intensity—were generally comparable across studies. There were no systematic differences in delivery setting (e.g., outpatient vs. inpatient care) or therapist qualifications that would violate the assumption of transitivity. The outcome measures were conceptually equivalent and commonly used interchangeably in musculoskeletal trials, thus permitting valid indirect comparisons. Collectively, these characteristics support the methodological integrity and clinical plausibility of transitivity within the NMA framework [21].

### 2.5. Methodological Quality Appraisal

The methodological quality of the included randomized trials was evaluated using the Cochrane Risk of Bias tool (Version 2, RoB 2, London, UK) [21]. This instrument assesses six key domains: the randomization process, deviations from intended interventions, missing outcome data, measurement of outcomes, selection of reported results, and overall risk of bias.

### 2.6. Primary Outcome: Functional Improvement (Standardized Mean Difference)

The primary outcome of this study is to compare the effectiveness of physiotherapy treatments in improving function in individuals with RCRSP. Functional outcomes are assessed using the Shoulder Pain and Disability Index (SPADI) and the Disabilities of the Arm, Shoulder, and Hand (DASH) Score. All scores are standardized to a 0–100 scale to ensure comparability across studies.

### 2.7. Secondary Outcomes: Pain Reduction (Standardized Mean Difference)

The secondary outcome is the effectiveness of physiotherapy treatments in reducing pain in individuals with RCRSP. Pain levels are assessed using the Visual Analog Scale (VAS) and the Numeric Pain Rating Scale (NPRS). The scores are standardized to a 0–10 scale for consistency and comparability across studies.

### 2.8. Data Extraction, Management, and Conversion

Two authors independently extracted data from the included studies, encompassing demographic characteristics, study design, assessment criteria, and both primary and secondary outcomes. When essential data were not available in the published reports, the corresponding authors were contacted to obtain the original information.

Data extraction, transformation, and synthesis were carried out in accordance with the guidelines provided in the Cochrane Handbook for Systematic Reviews of Interventions and other relevant medical literature [28,29,30,31,32].

For studies that did not report standard deviations (SDs) for continuous outcomes, SDs were imputed using established formulas based on available data such as confidence intervals, standard errors, or *p*-values. When such data were not available, we attempted to contact study authors. If the required information could not be retrieved or reliably estimated, the study was excluded from the quantitative synthesis to maintain analytical rigor and avoid introducing bias. Zero-event studies were not applicable in this review, as all included outcomes were continuous measures.

### 2.9. Statistical Analysis

Given the variety of physiotherapy interventions for RCRSP, a random-effects model was employed for the network meta-analysis [33]. The analysis was conducted using MetaInsight (Version 4.0.2, Complex Reviews Support Unit, National Institute for Health Research, London, UK) under a frequentist framework. MetaInsight is a web-based platform for network meta-analysis that utilizes the netmeta package in R for frequentist statistical calculations [22]

Forest and network plots were initially constructed to illustrate all pairwise comparisons across the included studies. Subsequently, forest plots were created to illustrate comparisons of standardized mean differences in evaluating the effectiveness of physiotherapy treatments for both functional improvement and pain reduction. Effect sizes were reported as point estimates with 95% CIs [34]. The effectiveness of physiotherapy treatments for functional improvement and pain reduction in RCRSP was ranked, and numerical results for both direct and indirect treatment comparisons are presented in the tables. Inconsistency was assessed to detect potential conflicts within the network. Statistical significance was defined as a two-tailed *p*-value of less than 0.05.

### 2.10. Sensitivity Analyses

A single-study deletion approach was employed to test result stability; each of the twelve studies was removed in turn. This sensitivity analysis confirmed that conclusions and rankings were not driven by any single study [32].

### 2.11. Publication Bias

Potential publication bias was assessed following the recommendations of the Cochrane Handbook for Systematic Reviews of Interventions [28]. A funnel plot was constructed using Comprehensive Meta-Analysis software (Version 4; Biostat, Englewood, NJ, USA), and Egger’s regression test was applied to assess asymmetry. These analyses were performed across all pairwise comparisons, using the most common comparator (e.g., control or exercise therapy) as the reference group. Visual inspection of the funnel plot revealed no major asymmetry, and Egger’s test did not indicate statistically significant publication bias.

## 3. Results

### 3.1. Study Identification and Network Model Formation

A PRISMA flowchart detailing the literature search is presented in Figure 1, and the PRISMA network meta-analysis extension checklist is provided in Table S1. From an initial pool of 29 articles, 11 RCTs (*n* = 548) met the inclusion criteria (Table 1) [3,11,12,13,14,15,16,35,36,37,38]. As summarized in Supplementary Table S3, the most frequent reasons for excluding 18 full-text articles [2,6,9,10,17,18,20,39,40,41,42,43,44,45,46,47,48,49] included a follow-up duration shorter than 12 weeks (*n* = 8), absence of a control group (*n* = 6), use of invasive interventions such as injections or surgery (*n* = 2), and missing key outcome data (*n* = 2). These criteria were applied consistently to ensure methodological comparability.

**Figure 2:**
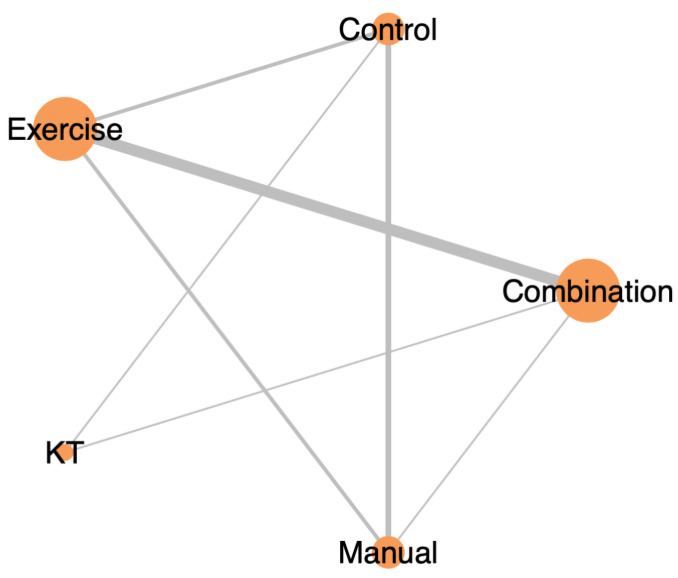
Network plot illustrating the direct comparisons between physiotherapy interventions for RCRSP in terms of pain reduction after 12 weeks. Node size reflects the number of participants per treatment, and edge thickness indicates the number of studies informing each comparison.

Among the included participants, 152 received exercise therapy alone, 66 received manual therapy alone, 78 received KT, 140 received combination therapy (e.g., exercise plus manual therapy, KT, or extracorporeal shock wave therapy (ESWT)), and 112 were assigned to control groups. These allocations are illustrated in the network plot (Figure 2), where node sizes reflect total sample size and edge thickness denotes the number of studies per direct comparison.

### 3.2. Methodological Quality of the Included Studies

In assessing the overall methodological quality, we found that 55% (6/11) of the studies had a low risk of bias, while 45% (5/11) exhibited some degree of bias (Supplementary Figure S1). Studies with potential risk of bias often showed variations in protocols between study arms, which may have influenced adherence and outcomes. Detailed findings from the risk of bias assessment are presented in Supplementary Table S4.

### 3.3. Primary Outcome: Combination Therapy Most Effective for Functional Improvement

**Figure 3:**
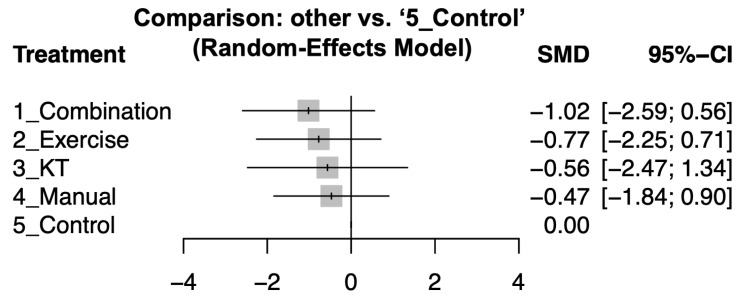
Forest plot showing how different treatments affected shoulder function compared to control after 12-week intervention. Negative values mean better improvement.

After a 12-week intervention, combination therapy showed significant functional improvement (effect size: −1.02; 95% CI: −2.59 to 0.56). In contrast, exercise therapy (effect size: −0.77; 95% CI: −2.25 to 0.71) and KT (effect size: −0.56; 95% CI: −2.47 to 1.34) produced modest effects. On the other hand, manual therapy (effect size: −0.47; 95% CI: −1.84 to 0.9) did not show a significant difference compared to the control group. Detailed pairwise comparisons between study arms are shown in Supplementary Figure S2 and summarized in Figure 3. Treatments were ranked based on their effect sizes for functional improvement, with combination therapy being the most effective, followed by exercise therapy, KT, and manual therapy. Please see Table 2 for a detailed comparison and ranking.

### 3.4. Secondary Outcome: Combination Therapy Most Effective for Pain Reduction

**Figure 4:**
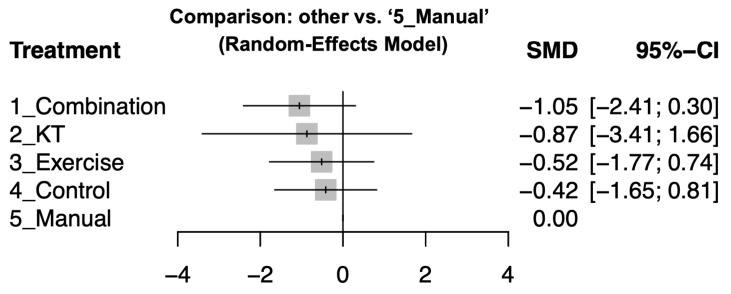
Funnel plot assessing publication bias in the meta-analysis.

After a 12-week intervention, combination therapy also showed significant pain reduction (effect size: −1.05; 95% CI: −2.41 to 0.3). KT (effect size: −0.87; 95% CI: −3.41 to 1.66) and exercise therapy (effect size: −0.52; 95% CI: −1.77 to 0.74) demonstrated marginal effects while manual therapy did not demonstrate a statistically significant improvement compared to the control groups. Detailed pairwise comparisons are illustrated in Supplementary Figure S3 and summarized in Figure 4. Treatments were ranked based on their effect sizes for pain reduction, with combination therapy being the most effective, followed by exercise therapy, KT, and manual therapy. Please see Table 3 for the complete comparison and ranking.

### 3.5. Inconsistency Test

Consistency between direct and indirect comparisons was assessed using a global inconsistency test within the frequentist framework. All comparisons yielded non-significant results (*p* > 0.05), indicating no evidence of inconsistency across the network. These findings suggest that the transitivity assumption holds and that the pooled estimates are reliable across mixed sources of evidence. Detailed results are provided in Supplementary Tables S5 and S6.

However, certain treatment comparisons—particularly manual therapy versus KT (*n* = 2) and manual therapy versus combination therapy (*n* = 1)—were informed by a limited number of studies and participants. These nodes may be underpowered, and the corresponding effect estimates should be interpreted with caution.

### 3.6. Sensitivity Analyses

To evaluate the influence of study quality on the robustness of our findings, we conducted two complementary sensitivity analyses.

First, a leave-one-out (one-study removal) analysis demonstrated consistent treatment rankings and effect size estimates across all iterations. Combination therapy remained the most effective intervention in terms of both functional improvement and pain reduction. Interventions such as exercise therapy, KT, and manual therapy showed marginal effects, with no changes in direction or statistical significance when any single study was removed. These results suggest that no individual study exerted disproportionate influence on the overall conclusions (Supplementary Figures S4a–k).

Second, we conducted a subgroup sensitivity analysis excluding five studies assessed as having either “some concerns” or “high risk” of bias based on the Cochrane Risk of Bias 2.0 tool. As shown in Supplementary Figure S5a–j, the overall treatment rankings and relative effect sizes remained largely unchanged. Combination therapy continued to outperform other interventions across both primary outcomes.

Together, these analyses reinforce the methodological robustness of our findings and indicate that the conclusions regarding the superiority of combination therapy are not materially affected by study-level bias or the inclusion of specific trials.

## 4. Discussion

This network meta-analysis synthesized data from 11 randomized controlled trials to compare the efficacy of different physiotherapy interventions for RCRSP. Our analysis demonstrated that combination therapy (exercise plus manual therapy) yielded superior improvements in both functional outcomes and pain reduction at the 12-week mark, compared with single-modality treatments [14,38]. These improvements exceeded the established minimal clinically important differences (MCIDs) for DASH, SPADI, and VAS/NPRS measures, confirming both statistical and clinical relevance. These comparisons are detailed in Supplementary Table S7 [50,51,52,53].

The superiority of combination therapy is consistent with prior network meta-analyses and systematic reviews [1,27,54]. Notably, single-modality interventions often target isolated mechanisms (e.g., strengthening or joint mobility), whereas combination approaches address the multifactorial pathophysiology of RCRSP, including muscular deficits, joint instability, and central sensitization [18,54]. The synergistic effects of exercise and manual therapy are likely underpinned by complementary mechanisms: exercise promotes neuromuscular adaptation and joint stability [11,16,35], while manual therapy provides immediate pain modulation via mechanoreceptor activation and descending inhibition pathways [12,18]. This synergy potentially enhances patient tolerance and engagement, improving adherence and outcomes [23].

Mechanistic hypotheses are supported by prior studies. Camargo et al. [20] further reported that specific exercise and manual therapy improved kinematic control and reduced symptom chronicity. Moreover, combining interventions likely amplifies cortical plasticity and descending nociceptive inhibition, as suggested in neurophysiological literature [36,37,38,40]. From a biopsychosocial standpoint, multimodal therapy may also positively influence patient motivation and therapeutic alliance, which are increasingly recognized as important determinants of musculoskeletal outcomes [23].

However, most previous studies primarily focused on short-term outcomes, reporting immediate improvements such as ≥30% reduction in VAS scores or significant improvements in DASH scores (>10 points) [53,55], without thorough examination of mid- to long-term effects [4,17]. Our study addressed this gap by employing a fixed 12-week intervention framework to evaluate mid-term outcomes, demonstrating that combination therapy resulted in clinically meaningful improvements in upper limb function, reaching the minimal clinically important difference (MCID) in DASH scores.

Furthermore, the literature has reported that the recurrence rate of shoulder pain is approximately 30–40%, and 10–15% of patients continue to experience pain and functional limitations 12 months post-treatment [1,6]. Nonetheless, many studies lack structured designs specifying intervention durations [56,57,58], making it difficult to establish optimal treatment protocols in clinical practice. By standardizing the 12-week intervention period, our study not only helped clarify the comparative effectiveness of different modalities but also emphasized the critical role of treatment duration and frequency in clinical outcomes.

From a clinical perspective, our findings suggest a dual-focus strategy for RCRSP management: an initial phase focused on pain relief (through manual and adjunctive therapies), followed by progressive neuromuscular strengthening of the rotator cuff and scapular stabilizers to promote long-term functional recovery and prevent recurrence. Treatment strategies should be individualized based on different disease stages (acute, subacute, chronic phases) to optimize outcomes. Although combination therapy was more effective, it may be harder to use in everyday practice. It can take more time and cost more, especially if manual therapy is needed at every visit. This might not be practical in busy clinics or for patients with limited access. Future studies should look at how realistic and cost-effective this treatment is in real-world settings.

### Study Limitations

This study has several limitations that warrant consideration. First, although no language restrictions were applied during the literature search, all included trials were published in English, introducing potential language bias. Future systematic reviews should incorporate non-English databases and multilingual collaboration to enhance comprehensiveness.

Second, selective reporting bias may have been present in trials lacking protocol registration or complete outcome disclosure. We recommend that future studies ensure transparent reporting and pre-registration of protocols to strengthen evidence reliability.

Third, variability in therapist skill and treatment fidelity across studies may have introduced indirect bias. Differences in clinician experience, training, and adherence to standardized protocols can influence treatment outcomes. Future trials should report provider qualifications and consider stratifying analyses by provider consistency to improve replicability.

Fourth, this review focused on outcomes measured at 12 weeks to ensure consistency across trials. However, this limits the ability to evaluate long-term treatment durability. Given the 30–40% recurrence rate of RCRSP and persistent symptoms in up to 15% of patients after one year, future studies should incorporate longer follow-up periods (e.g., 6–12 months) to assess sustainability and recurrence.

Fifth, although combination therapy demonstrated favorable point estimates, some comparisons yielded wide confidence intervals that crossed the null (e.g., −2.59 to 0.56 for function), indicating imprecision and statistical uncertainty. This suggests that the true effect may be smaller or inconclusive, emphasizing the need for larger, well-powered trials to validate these findings with greater precision.

Sixth, we did not explore how patient characteristics such as age or chronicity might influence treatment outcomes. This limits our ability to identify which groups respond best. Future research should examine these factors to support more personalized treatment plans.

Finally, although interventions were grouped by type (e.g., exercise, manual therapy), substantial heterogeneity existed in dosage, frequency, and technique. This may affect comparability and generalizability. Greater standardization and clearer reporting of physiotherapy protocols are essential for improving future evidence synthesis.

## 5. Conclusions

Combination therapy, especially exercise plus manual therapy, showed the greatest improvements in pain and function for RCRSP, with effect sizes exceeding clinical thresholds. These results support its use in practice. To enhance comparability and clinical translation, future trials should adopt standardized intervention protocols and longer follow-up durations.

## Figures and Tables

**Table 1:** Summary of included trials evaluating physiotherapy interventions for RCRSP to improve shoulder function and reduce pain.

First Author and Year	Enrolled Population (Group population)	Population in Nodes	12-Week DASH Improvement	12-Week VAS Reduction	Summary
Aytar, 2015 [12]	Subacromial impingement syndrome; Total 66 (Manual: 22, Control:22, Exercise:22)	Manual Control Exercise	−12.7 ± 14.2 −17.4 ± 11.5 −17.0 ± 10.4	−3.0 ± 2.5 −1.4 ± 1.5 −2.5 ± 1.7	This randomized controlled trial compared scapular mobilization, sham mobilization, and supervised exercise for subacromial impingement syndrome (SAIS). All groups improved similarly in shoulder function, pain, and range of motion, without significant differences between interventions. Scapular mobilization showed no additional benefit over supervised exercise or sham treatment in managing SAIS symptoms.
Arias-Buría, 2017 [36]	Subacromial pain syndrome; Total: 50 (Exercise: 25, Exercise+ TrP-DN: 25)	Combination (Exercise + TrP-DN) Exercise	−50.7 ± 4.1 −28.2 ± 7.4	−3.4 ± 0.9 −3.2 ± 0.9	This trial assessed the effectiveness of adding Trigger Point Dry Needling (TrP-DN) to exercises in patients with SAPS. Results indicated both groups improved in pain, but the exercise plus TrP-DN group showed significantly greater improvements in shoulder-related disability across all follow-ups (3, 6, and 12 months), demonstrating sustained functional benefits.
Gunay-Ucurum, 2018 [38]	Subacromial pain syndrome; Total: 39 (Interferential + Exercise: 20, Exercise: 19)	Combination (Interferential + Exercise) Exercise	−24.1 ± 13.0 −18.4 ± 14.0	−1.5 ± 1.9 −1.9 ± 1.7	This randomized controlled trial evaluated exercise therapy combined with electrotherapy modalities in patients with shoulder impingement syndrome. All groups improved significantly in pain, function, and physical quality of life at 3 months, with interferential current providing additional significant benefits in mental quality of life compared to others.
Frassanito, 2018 [13]	Rotator cuff calcific tendinopathy; Total: 42 (ESWT + KT: 21, ESWT:21)	Combination (ESWT + KT) Kinesio Taping	−23.6 ± 5.4 −27.9 ± 6.1	−2.1 ± 0.9 −2.8 ± 1.1	This randomized controlled trial assessed whether combining Extracorporeal Shock Wave Therapy (ESWT) with Kinesio Taping (KT) is superior to ESWT alone for rotator cuff calcific tendinopathy. Forty-two patients participated, showing that ESWT combined with KT provided significantly faster pain relief and improved shoulder function compared to ESWT alone, with sustained benefits at 12 weeks post-treatment.
Gutiérrez-Espinoza, 2019 [11]	Subacromial pain syndrome; Total: 80 (Exercise + Manual: 40, Exercise: 40)	Combination (Exercise + Manual) Exercise	−18.5 ± 7.3 −23.9 ± 7.3	−3.3 ± 1.1 −2.9 ± 1.1	This randomized controlled trial compared an exercise program alone versus an exercise program combined with pectoralis minor stretching for subacromial pain syndrome (SPS). Results showed no significant short-term clinical benefits in function, pain, or muscle length with the addition of pectoralis minor stretching to the exercise program in SPS participants.
İğrek, 2022 [35]	Subacromial Impingement Syndrome; Total: 44 (Control: 14, SM: 15, PNF: 15)	Exercise Manual Control	−46.4 ± 14.6 −50.1 ± 14.7 −37.6 ± 11.0	−3.2 ± 1.3 −3.0 ± 0.9 −3.3 ± 0.9	This randomized clinical trial compared proprioceptive neuromuscular facilitation (PNF) and shoulder mobilization (SM) added to conventional physiotherapy for SIS. Both treatments improved pain and functionality better than conventional therapy alone. SM showed greater benefits in shoulder flexion range of motion, while PNF improved shoulder extension strength more effectively.
Hunter, 2022 [3]	Shoulder impingement syndrome; Total 50 (MET + Soft tissue massage: 25, Control: 25)	Manual (MET + Soft tissue massage) Control	−13.4 ± 10.5 −29.6 ± 8.7	−0.5 ± 1.3 −2.1 ± 1.1	This trial compared the thoracic spine muscle energy technique (MET) plus soft tissue massage and placebo in patients with SIS. MET significantly reduced shoulder pain and disability at 6 and 12 months compared to placebo, suggesting thoracic manual therapy as beneficial for long-term SIS management.
de Oliveira 2022 [15]	Subacromial pain syndrome; Total 24 (BioFB+ Exercise: 12, Exercise: 12)	Combination (BioFB + Exercise) Exercise	−22.5 ± 8.1 −8.5 ± 6.3	−4.6 ± 1.9 −4.4 ± 1.8	This trial compared exercises alone versus exercises with electromyographic biofeedback for subacromial pain syndrome (SPS). Both groups improved pain and function; however, adding biofeedback did not further enhance pain relief or function. Biofeedback significantly increased scapular upward rotation but did not provide superior outcomes in strength, range of motion, or muscle activation.
Umay-Altaş, 2023 [14]	Subacromial pain syndrome; Total 60 (Exercise + KT: 30 Exercise: 30)	Combination (Exercise + KT) Exercise	−22.4 ± 5.9 −15.5 ± 5.3	−4.6 ± 1.1 −0.8 ± 1.3	This randomized controlled study compared a Kinesio Taping (KT) method in female SIS patients. The KT applications improved pain, function, grip strength, acromiohumeral distance (AHD), and supraspinatus tendon thickness versus control. Taping on the deltoid muscle provided superior short- and mid-term improvements, particularly for pain, functional status, and AHD, supporting its clinical effectiveness.
Nazary-Moghadam, 2024 [16]	Subacromial Pain Syndrome; Total: 40 (CPT: 20, RPT: 20)	Combination (Exercise + Manual) Exercise	−31.3 ± 13.71 −8.3 ± 10.64	−1.7 ± 1.4 −3.8 ± 1.1	This randomized trial compared comprehensive physiotherapy (CPT), including scapular exercises and thoracic mobilization, with routine physiotherapy (RPT) for subacromial pain syndrome. Both groups improved immediately, but at 6 months, CPT significantly outperformed RPT in reducing pain, improving shoulder function, and enhancing quality of life, suggesting sustained long-term benefits with the comprehensive approach.
Valenzuela-Rios, 2024 [37]	Subacromial pain syndrome; Total 39 (PENS+ Exercise: 20 Exercise: 19)	Combination (PENS+ Exercise) Exercise	−25.0 ± 14.1 −17.3 ± 12.7	−2.5 ± 0.9 −3.1 ± 1.4	This trial compared exercise alone, exercise with percutaneous electrical nerve stimulation (PENS), and exercise with placebo PENS for SAPS. All groups improved in disability and psychological outcomes. Adding PENS provided a small, short-term advantage only in shoulder pain reduction at one month; however, there were no significant differences in disability or psychological outcomes.

ESWT: extracorporeal shock wave therapy; KT: Kinesio taping; MET: muscle energy technique; SAIS: subacromial impingement syndrome; PNF: proprioceptive neuromuscular facilitation; SM: shoulder mobilization; TrP-DN: trigger point dry needling; SIS: shoulder impingement syndrome; SPS: subacromial pain syndrome; PENS: percutaneous electrical nerve stimulation.

**Table 2:** Pairwise comparison and ranking of different therapy types of interventions for improvement of shoulder function at 12 weeks in rotator cuff-related shoulder pain.

**Combination**	−0.08 [−1.10; 0.93]	−0.11 [−2.57; 2.36]	−1.87 [−4.38; 0.63]	.
−0.24 [−1.19; 0.70]	**Exercise**	.	−0.04 [−1.83; 1.75]	−0.31 [−2.09; 1.47]
−0.45 [−2.36; 1.46]	−0.21 [−2.20; 1.78]	**KT**	.	−0.22 [−2.68; 2.23]
−0.55 [−2.04; 0.94]	−0.30 [−1.72; 1.12]	−0.09 [−2.21; 2.02]	**Manual**	−0.76 [−2.21; 0.70]
−1.02 [−2.59; 0.56]	−0.77 [−2.25; 0.71]	−0.56 [−2.47; 1.34]	−0.47 [−1.84; 0.90]	**Control**

The estimates from pairwise meta-analyses are situated above the diagonal line, while the estimates from the network meta-analyses are located below the diagonal line.

**Table 3:** Pairwise comparison and ranking of different therapy types of interventions for reduction of shoulder pain at 12 weeks in rotator cuff-related shoulder pain.

**Combination**	−0.18 [−2.32; 1.96]	−0.45 [−1.33; 0.42]	.	−1.56 [−3.73; 0.61]
−0.18 [−2.32; 1.96]	**KT**	.	.	.
−0.54 [−1.37; 0.30]	−0.36 [−2.66; 1.94]	**Exercise**	−0.24 [−1.79; 1.31]	0.03 [−1.53; 1.60]
−0.64 [−2.17; 0.90]	−0.46 [−3.09; 2.18]	−0.10 [−1.49; 1.30]	**Control**	−0.33 [−1.59; 0.94]
−1.05 [−2.41; 0.30]	−0.87 [−3.41; 1.66]	−0.52 [−1.77; 0.74]	−0.42 [−1.65; 0.81]	**Manual**

The estimates from pairwise meta-analyses are situated above the diagonal line, while the estimates from the network meta-analyses are located below the diagonal line.

## Data Availability

All related data are included and no new data were created.

## Supplementary Materials

The following supporting information can be downloaded at https://www.mdpi.com/article/10.3390/jcm14134765/s1.

## Abbreviations

The following abbreviations are used in this manuscript:

RCRSP	Rotator cuff-related shoulder pain
NMA	Meta-analysis
PRISMA	Preferred Reporting Items for Systematic Reviews and Meta-Analyses
PRISMA-NMA	PRISMA extension guidelines for network meta-analysis
KT	Kinesio taping
SPADI	Shoulder Pain and Disability Index
DASH	Disabilities of the Arm, Shoulder and Hand
VAS	Visual Analog Scale
NPRS	Numeric Pain Rating Scale
SIS	Subacromial impingement syndrome
TENS	Transcutaneous electrical nerve stimulation
EMS	Electrical muscular stimulation
PNF	Proprioceptive neuromuscular facilitation
SM	Shoulder mobilization
TrP-DN	Trigger point dry needling
SPS	Subacromial pain syndrome
PENS	Percutaneous electrical nerve stimulation
ESWT	Extracorporeal shock wave therapy
MET	Muscle energy technique
