# Supplementary material for: Comparative Effectiveness of Combination Versus Single-Modality Physiotherapy for Rotator Cuff-Related Shoulder Pain: A Systematic Review and Network Meta-Analysis
Source: J Clin Med. 2025 Jul 5;14(13):4765. doi: 10.3390/jcm14134765 (PMC12250685; doi:10.3390/jcm14134765)
Supplement: Supplementary file 1 [file jcm-14-04765-s001.zip › TableS2_keyword_search.pdf]

Table S2 - Keywords and search results in different databases

Database	Keyword	Filter	Date	Results
PubMed	('Rotator cuff related shoulder pain' or 'RCRSP') AND ('subacromial impingement syndrome' or 'SBPS') AND ('shoulder impingement syndrome ' or 'SIS') AND ('physical therapy ' or 'rehabilitation ') AND ('Pain' OR 'Vas') AND ('DASH ' OR 'Disabilities of the Arm, Shoulder, and Hand ')	NA	April 7, 2025	370
Embase	('Rotator cuff related shoulder pain' or 'RCRSP') AND ('subacromial impingement syndrome' or 'SBPS') AND ('shoulder impingement syndrome ' or 'SIS') AND ('physical therapy ' or 'rehabilitation ') AND ('Pain' OR 'Vas') AND ('DASH ' OR 'Disabilities of the Arm, Shoulder, and Hand ')	NA	April 7, 2025	355
Cochrane CENTRAL	('Rotator cuff related shoulder pain' or 'RCRSP') AND ('subacromial impingement syndrome' or 'SBPS') AND ('shoulder impingement syndrome ' or 'SIS') AND ('physical therapy ' or 'rehabilitation ') AND ('Pain' OR 'Vas') AND ('DASH ' OR 'Disabilities of the Arm, Shoulder, and Hand ')	Title Abstract Keyword	April 7, 2025	309
Cochrane Reviews	('Rotator cuff related shoulder pain' or 'RCRSP') AND ('subacromial impingement syndrome' or 'SBPS') AND ('shoulder impingement syndrome ' or 'SIS') AND ('physical therapy ' or 'rehabilitation ') AND ('Pain' OR 'Vas') AND ('DASH ' OR 'Disabilities of the Arm, Shoulder, and Hand ')	Title Abstract Keyword	April 7, 2025	10
ClinicalTrials.gov	('Rotator cuff related shoulder pain' or 'RCRSP') AND ('subacromial impingement syndrome' or 'SBPS') AND ('shoulder impingement syndrome ' or 'SIS') AND ('physical therapy ' or 'rehabilitation ') AND ('Pain' OR 'Vas') AND ('DASH ' OR 'Disabilities of the Arm, Shoulder, and Hand ')	Condition or disease	April 7, 2025	0

NA: not applied
