# Supplementary material for: Comparative Effectiveness of Combination Versus Single-Modality Physiotherapy for Rotator Cuff-Related Shoulder Pain: A Systematic Review and Network Meta-Analysis
Source: J Clin Med. 2025 Jul 5;14(13):4765. doi: 10.3390/jcm14134765 (PMC12250685; doi:10.3390/jcm14134765)
Supplement: Supplementary file 1 [file jcm-14-04765-s001.zip › TableS3_exclusion_reasons.pdf]

Table S3 - Excluded studies and reasons

Citations		Reasons
1.	Moslehi M, Letafatkar A, Miri H. Feedback improves the scapular-focused treatment effects in patients with shoulder impingement syndrome. Knee Surg Sports Traumatol Arthrosc. 2021;29:2281-2288.	Less than 12 weeks studies. Only 8 weeks data.
2.	Karamanlioglu DS, Kaysin MY, Begoglu FA, Akpinar P, Unlu Ozkan F, Aktas I. Effects of acupuncture on pain and function in patients with subacromial impingement syndrome: A randomized sham-controlled trial. Integr Med Res. 2024;13:101049.	Less than 12 weeks studies. Only 1 month data.
3.	Camargo PR, Alburquerque-Sendín F, Avila MA, Haik MN, Vieira A, Salvini TF. Effects of stretching and strengthening exercises, with and without manual therapy, on scapular kinematics, function, and pain in individuals with shoulder impingement: A randomized controlled trial. J Orthop Sports Phys Ther. 2015;45(12):984-997.	Less than 12 weeks studies. Only 4 weeks data.
4.	Arias-Buría JL, Truyols-Domínguez S, Valero-Alcaide R, Salom-Moreno J, Atín-Arratibel MA, Fernández-de-las-Peñas C. Ultrasound-guided percutaneous electrolysis and eccentric exercises for subacromial pain syndrome: A randomized clinical trial. Evid Based Complement Alternat Med. 2015;2015:315219.	Less than 12 weeks studies. Only 1 week data.
5.	Pérez-Merino L, Casajuana MC, Bernal G, et al. Evaluation of the effectiveness of three physiotherapeutic treatments for subacromial impingement syndrome: A randomised clinical trial. Physiotherapy. 2016;102(1):57-63.	Less than 12 weeks studies. Only 1 month data.
6.	Ager AL, Roy JS, Gamache F, Hébert LJ. The effectiveness of an upper extremity neuromuscular training program on the shoulder function of military members with a rotator cuff tendinopathy: A pilot randomized controlled trial. Mil Med. 2019;184(5-6):e385-e392.	Less than 12 weeks studies. Only 6 weeks data.
7.	Boudreau N, Gaudreault N, Roy JS, Bédard S, Balg F. The addition of glenohumeral adductor coactivation to a rotator cuff exercise program for rotator cuff tendinopathy: A single-blind randomized controlled trial. J Orthop Sports Phys Ther. 2019;49(3):126-135.	Less than 12 weeks studies. Only 6 weeks data.
8.	Menek B, Tarakci D, Tarakci E, Yilmaz Menek M. Investigation on the efficiency of the closed kinetic chain and video-based game exercise programs in the rotator cuff rupture: A randomized trial. Games Health J. 2022;11(5):298-306.	Less than 12 weeks studies. Only 6 weeks data.
9.	Menek B, Tarakci D, Algun ZC. The effect of Mulligan mobilization on pain and life quality of patients with rotator cuff syndrome: A randomized controlled trial. J Back Musculoskelet Rehabil. 2019;32(1):171-178.	Less than 12 weeks studies. Only post-treatment data.
10.	Ingwersen KG, Jensen SL, Sørensen L, Jørgensen HR, Christensen R, Søgaard K, Juul-Kristensen B. Three months of progressive high-load versus traditional low-load strength training among patients with rotator cuff tendinopathy: Primary results from the double-blind randomized controlled RoCTEx trial. Orthop J Sports Med. 2017;5(8):2325967117723292.	No Control groups. Only Exercise treatment data.
11.	Blume C, Wang-Price S, Trudelle-Jackson E, Ortiz A. Comparison of eccentric and concentric exercise interventions in adults with subacromial impingement syndrome. Int J Sports Phys Ther. 2015;10(4):441-455.	No Control groups. Only Exercise treatment data.
12.	Christiansen DH, Hjort J. Group-based exercise, individually supervised exercise and home-based exercise have similar clinical effects and cost-effectiveness in people with subacromial pain: A randomised trial. J Physiother. 2021;67(2):124-131.	No Control groups. Only Exercise treatment data.

13.	AlAnazi A, Alghadir AH, Gabr SA. Handgrip strength exercises modulate shoulder pain, function, and strength of rotator cuff muscles of patients with primary subacromial impingement syndrome. Biomed Res Int. 2022;2022:9151831.	No Control groups. Only Exercise treatment data.
14.	Çelik EB, Tuncer A. Comparing the efficacy of manual therapy and exercise to synchronized telerehabilitation with self-manual therapy and exercise in treating subacromial pain syndrome: A randomized controlled trial. Healthcare. 2024;12:1074.	No Control groups. Only Exercise treatment data.
15.	Raeesi J, Negahban H, Kachooei AR, Moradi A, Ebrahimzadeh MH, Daghiani M. Comparing the effect of physiotherapy and physiotherapy plus corticosteroid injection on pain intensity, disability, quality of life, and treatment effectiveness in patients with Subacromial Pain Syndrome: A randomized controlled trial. Disabil Rehabil. 2023;45(25):4218-4226.	Invasive Treatments studies.
16.	Daghiani M, Negahban H, Ebrahimzadeh MH, et al. The effectiveness of comprehensive physiotherapy compared with corticosteroid injection on pain, disability, treatment effectiveness, and quality of life in patients with subacromial pain syndrome: A parallel, single-blind, randomized controlled trial. Physiother Theory Pract. 2023;39(8):1591-1605.	Invasive Treatments studies.
17.	de Oliveira FCL, Pairot de Fontenay B, Bouyer LJ, Desmeules F, Roy JS. Kinesiotaping for the rehabilitation of rotator cuff–related shoulder pain: A randomized clinical trial. Sports Health. 2021;13(2):161-172.	No Pain scale data.
18.	Dubé M-O, Desmeules F, Lewis JS, Roy J-S. Does the addition of motor control or strengthening exercises to education result in better outcomes for rotator cuff-related shoulder pain? A multiarm randomised controlled trial. Br J Sports Med. 2023.	No Pain scale data.
