# Supplementary material for: Comparative Effectiveness of Combination Versus Single-Modality Physiotherapy for Rotator Cuff-Related Shoulder Pain: A Systematic Review and Network Meta-Analysis
Source: J Clin Med. 2025 Jul 5;14(13):4765. doi: 10.3390/jcm14134765 (PMC12250685; doi:10.3390/jcm14134765)
Supplement: Supplementary file 1 [file jcm-14-04765-s001.zip › TableS4_FigS1_RoB2.pdf]

Table S4 - Detailed quality assessment of included studies using Cochrane risk of bias 2 tool

First author & Year	Randomization process	Intervention adherence	Missing outcome data	Outcome measurement	Selective reporting	Overall RoB
2015 Aytar	L	L	L	L	L	L
2017 Arias-Buría	L	S	L	L	L	S
2018 Frassanito	L	L	L	L	L	L
2018 Gunay- Ucurum	L	S	L	L	L	S
2019 Gutiérrez-Espiza	L	S	L	L	L	S
2022 İğrek	L	L	L	L	L	L
2022 Hunter	L	S	L	L	L	S
2022 Azevedo de, Oliveira	L	L	L	L	L	L
2022 Umay-Altaş	L	S	L	L	L	S
2024 Valenzuela-Rios	L	L	L	L	L	L
2024 Nazary-Moghadam	L	L	L	L	L	L

H, high risk of bias; L, low risk of bias; S, some risk of bias.

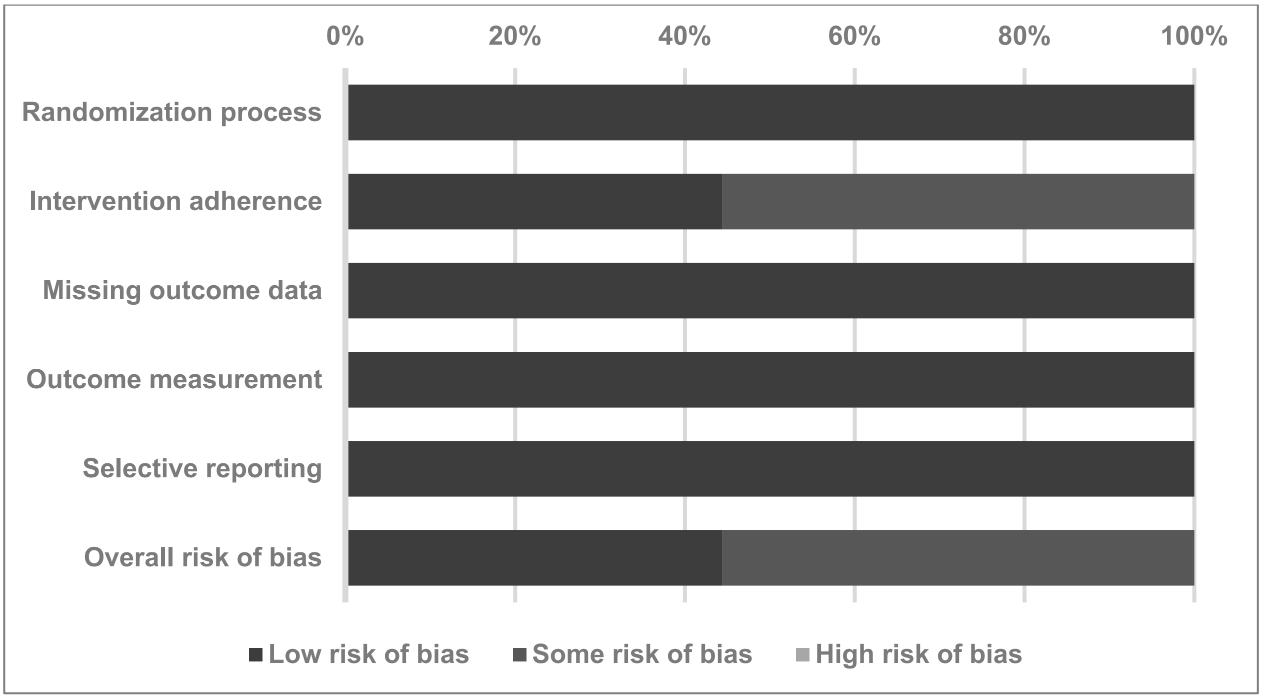

Figure S1. Summary of quality assessment of studies included in the network meta-analysis using Cochrane risk of bias 2 tool
