# Supplementary material for: Comparative Effectiveness of Combination Versus Single-Modality Physiotherapy for Rotator Cuff-Related Shoulder Pain: A Systematic Review and Network Meta-Analysis
Source: J Clin Med. 2025 Jul 5;14(13):4765. doi: 10.3390/jcm14134765 (PMC12250685; doi:10.3390/jcm14134765)
Supplement: Supplementary file 1 [file jcm-14-04765-s001.zip › TableS5.pdf]

TableS5. Inconsistent test results of the standardized mean difference in shoulder function improvement for rotator cuff-related shoulder pain after 12 weeks of therapy types.

**Comparison**	**Studies**	**NMA**	**Direct**	**Indirect**	**Difference**	**95CIL**	**95CIU**	***p* value**
Combination:Control	0	-1.1	NA	-1.1	NA	NA	NA	NA
Combination:Exercise	6	-0.2	-0.1	-1.3	1.2	-1.6	3.9	0.4
Combination: KT	1	-0.4	-0.1	-0.9	0.9	-3.1	4.8	0.7
Combination:Manual	1	-0.5	-1.9	0.2	-2.1	-5.2	1.1	0.2
Exercise: Control	2	-0.7	-0.3	-1.8	1.5	-1.7	4.7	0.4
KT: Control	1	-0.5	-0.2	-1.1	0.9	-3.1	4.8	0.7
Manual: Control	3	-0.4	-0.7	1.9	-2.6	-7.1	1.8	0.2
Exercise: KT	0	-0.2	NA	-0.2	NA	NA	NA	NA
Exercise: Manual	2	-0.3	-0.1	-0.8	0.7	-2.2	3.7	0.6
KT: Manual	0	-0.1	NA	-0.1	NA	NA	NA	NA

95CIL: lower limit of 95% confidence interval; 95CIU: upper limit of 95% confidence interval; NMA: network meta-analysis
