# Supplementary material for: Comparative Effectiveness of Combination Versus Single-Modality Physiotherapy for Rotator Cuff-Related Shoulder Pain: A Systematic Review and Network Meta-Analysis
Source: J Clin Med. 2025 Jul 5;14(13):4765. doi: 10.3390/jcm14134765 (PMC12250685; doi:10.3390/jcm14134765)
Supplement: Supplementary file 1 [file jcm-14-04765-s001.zip › TableS6.pdf]

TableS6. Inconsistent test results of the standardized mean difference in shoulder pain reduction for rotator cuff-related shoulder pain after 12 weeks of therapy types.

**Comparison**	**Studies**	**NMA**	**Direct**	**Indirect**	**Difference**	**95CIL**	**95CIU**	***p* value**
Combination: Control	0	-0.6	NA	-0.6	NA	NA	NA	NA
Combination: Exercise	6	-0.5	-0.5	-1.3	0.8	-1.9	3.6	0.6
Combination: KT	1	-0.2	-0.2	NA	NA	NA	NA	NA
Combination: Manual	1	-1.1	-1.6	-0.7	-0.8	-3.6	1.9	0.6
Exercise: Control	2	-0.1	-0.2	0.5	-0.7	-4.3	2.8	0.7
KT: Control	0	-0.5	NA	-0.5	NA	NA	NA	NA
Manual: Control	3	0.4	0.3	1.9	-1.6	-7.1	3.8	0.6
Exercise: KT	0	0.4	NA	0.4	NA	NA	NA	NA
Exercise: Manual	2	-0.5	0.1	-1.5	1.6	-1.1	4.2	0.2
KT: Manual	0	-0.9	NA	-0.9	NA	NA	NA	NA

95CIL: lower limit of 95% confidence interval; 95CIU: upper limit of 95% confidence interval; NMA: network meta-analysis
