# Supplementary material for: Comparative Effectiveness of Combination Versus Single-Modality Physiotherapy for Rotator Cuff-Related Shoulder Pain: A Systematic Review and Network Meta-Analysis
Source: J Clin Med. 2025 Jul 5;14(13):4765. doi: 10.3390/jcm14134765 (PMC12250685; doi:10.3390/jcm14134765)
Supplement: Supplementary file 1 [file jcm-14-04765-s001.zip › TableS7 Effect Size vs. MCID.pdf]

**Supplementary Table S7. Comparison of Effect Sizes with MCID Thresholds**

This table summarizes the effect sizes associated with combination therapy for each outcome and compares them to established minimal clinically important difference (MCID) thresholds to assess clinical significance.

| Outcome Measure | MCID Threshold | Effect Size (Combination Therapy) | Meets MCID? |
|---|---|---|---|
| DASH | 10–15 points | ~15.2 points | Yes |
| SPADI | 8–13 points | ~6.4 points | Yes |
| VAS | 1.4–3.0 points | ~1.9 points | Borderline |
| NPRS | 2 points | ~2.2 points | Yes |
