# Supplementary figures and images for: Comparative Effectiveness of Combination Versus Single-Modality Physiotherapy for Rotator Cuff-Related Shoulder Pain: A Systematic Review and Network Meta-Analysis
Source: J Clin Med. 2025 Jul 5;14(13):4765. doi: 10.3390/jcm14134765 (PMC12250685; doi:10.3390/jcm14134765)

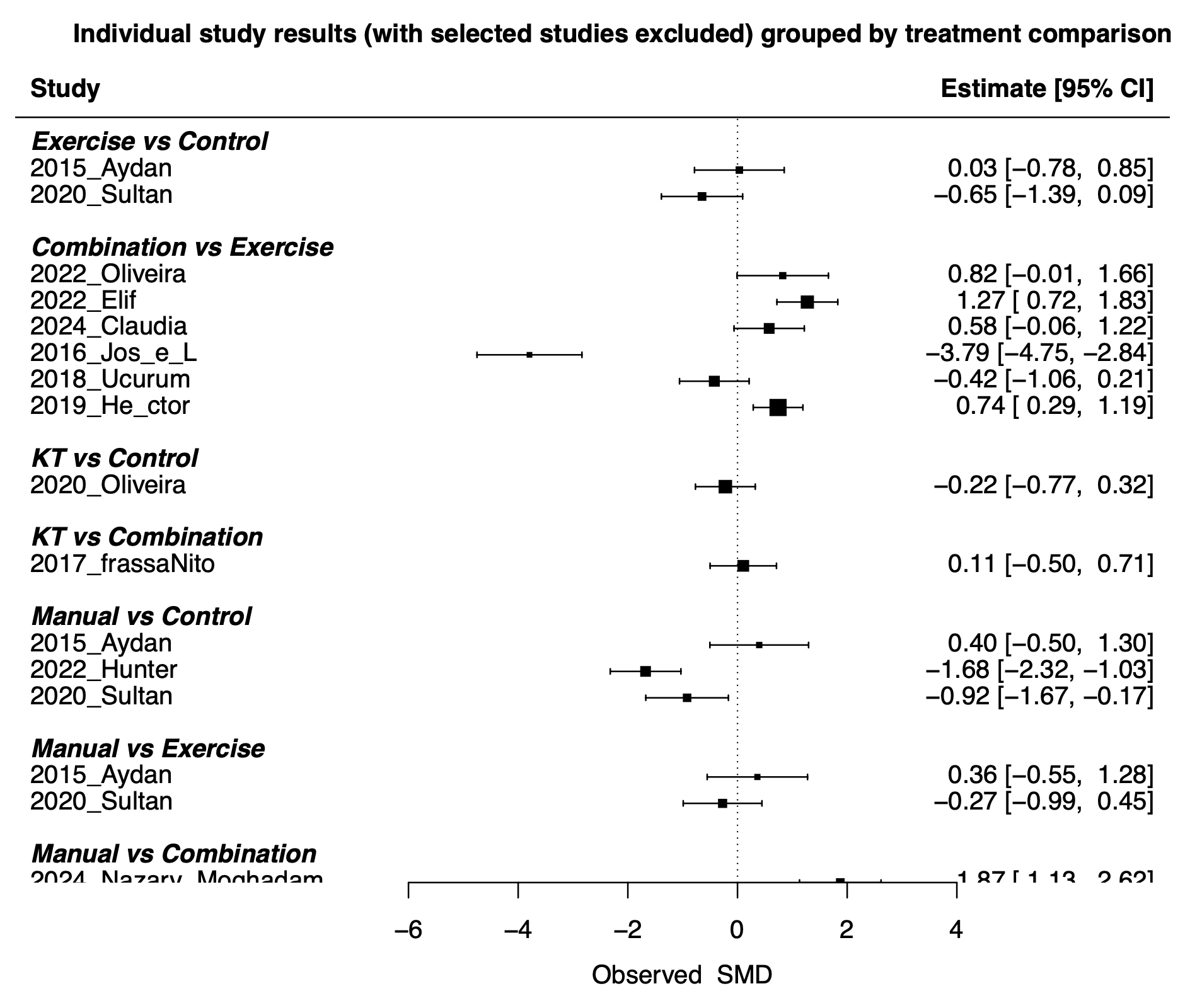

Supplement: Supplementary file 1 [file jcm-14-04765-s001.zip › FigS2.tiff]

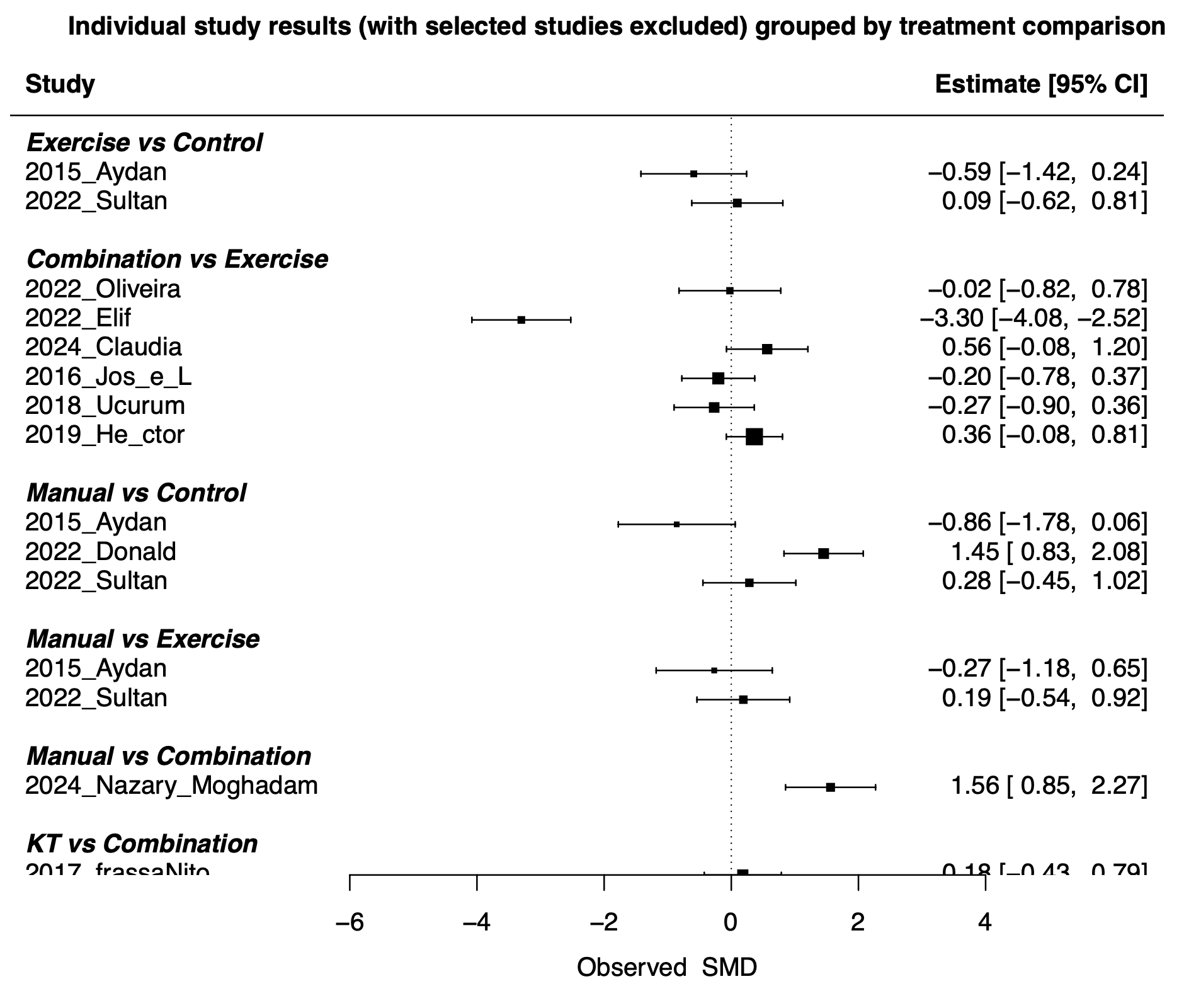

Supplement: Supplementary file 1 [file jcm-14-04765-s001.zip › FigS3.tiff]

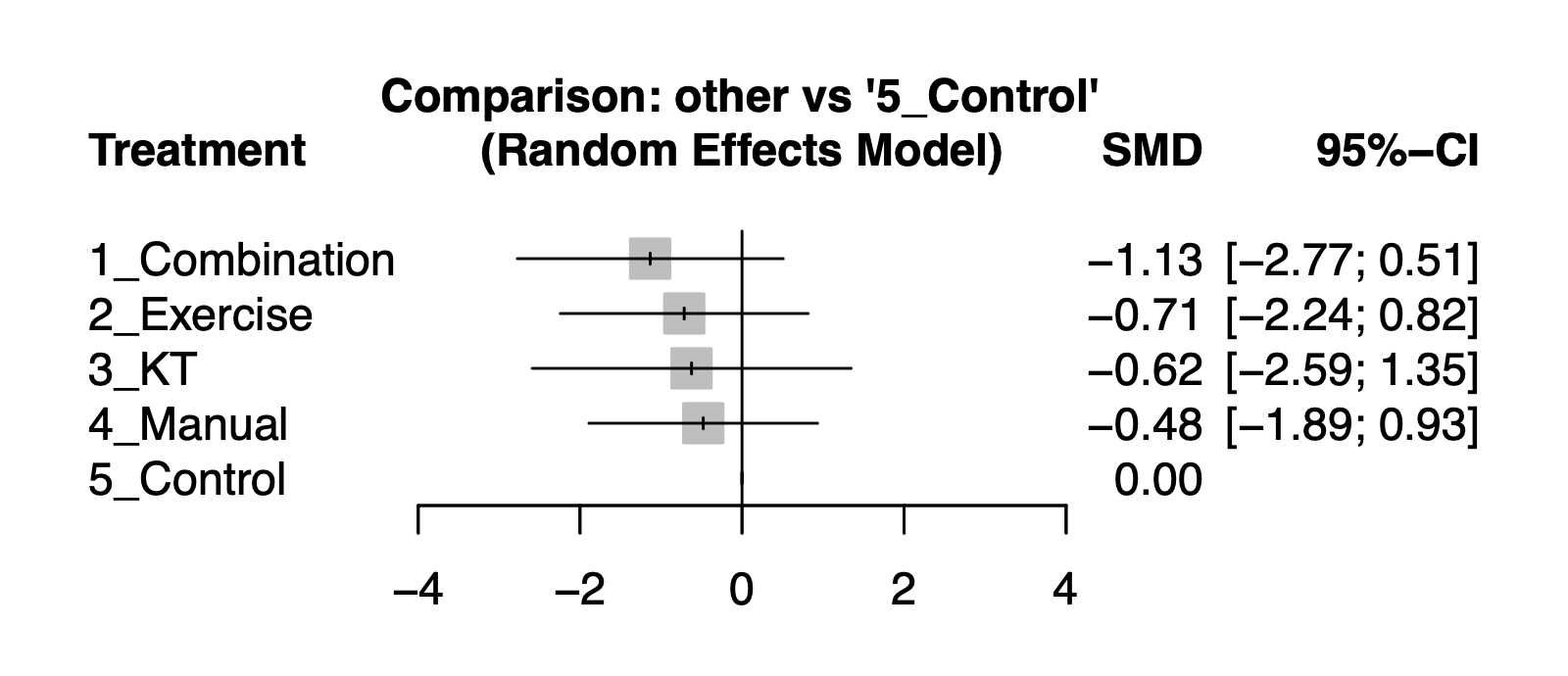

Supplement: Supplementary file 1 [file jcm-14-04765-s001.zip › FigS4a.tiff]

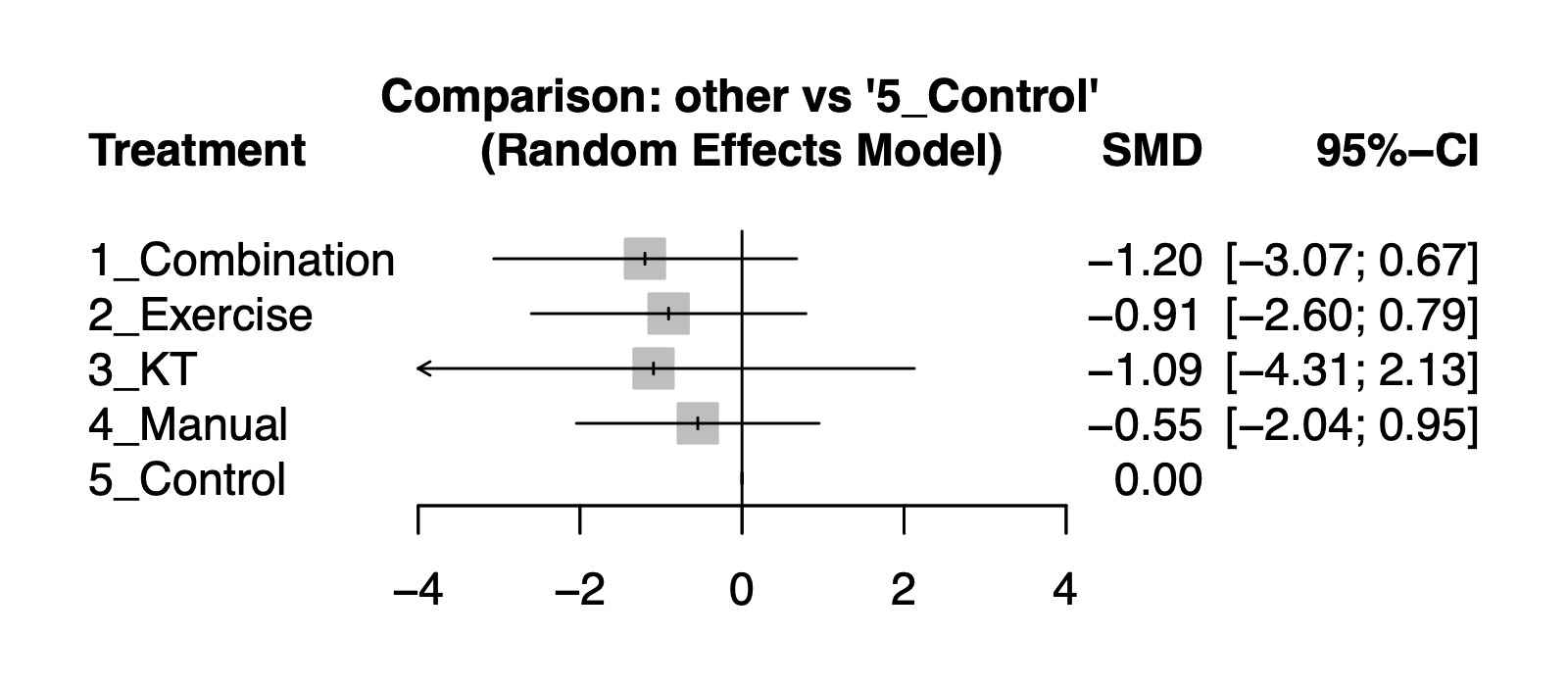

Supplement: Supplementary file 1 [file jcm-14-04765-s001.zip › FigS4b.tiff]

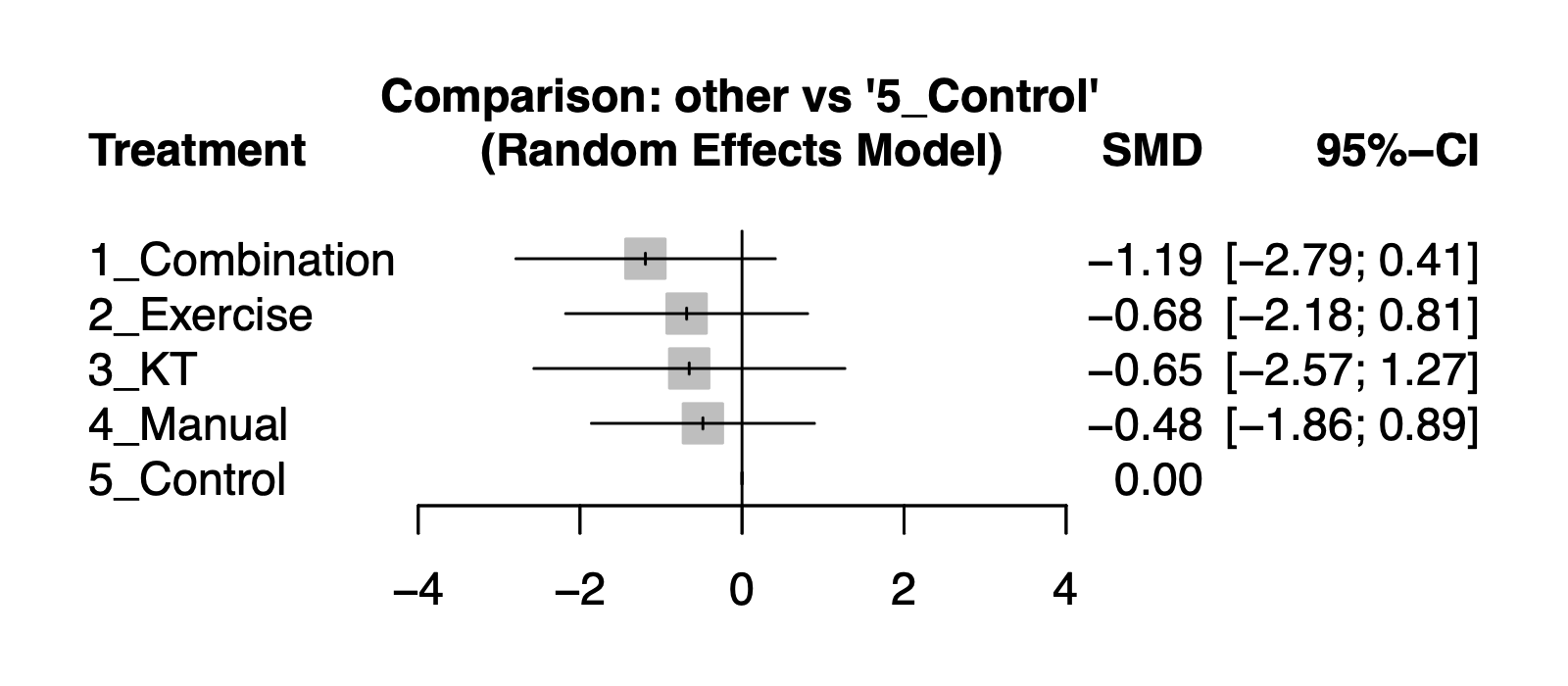

Supplement: Supplementary file 1 [file jcm-14-04765-s001.zip › FigS4c.tiff]

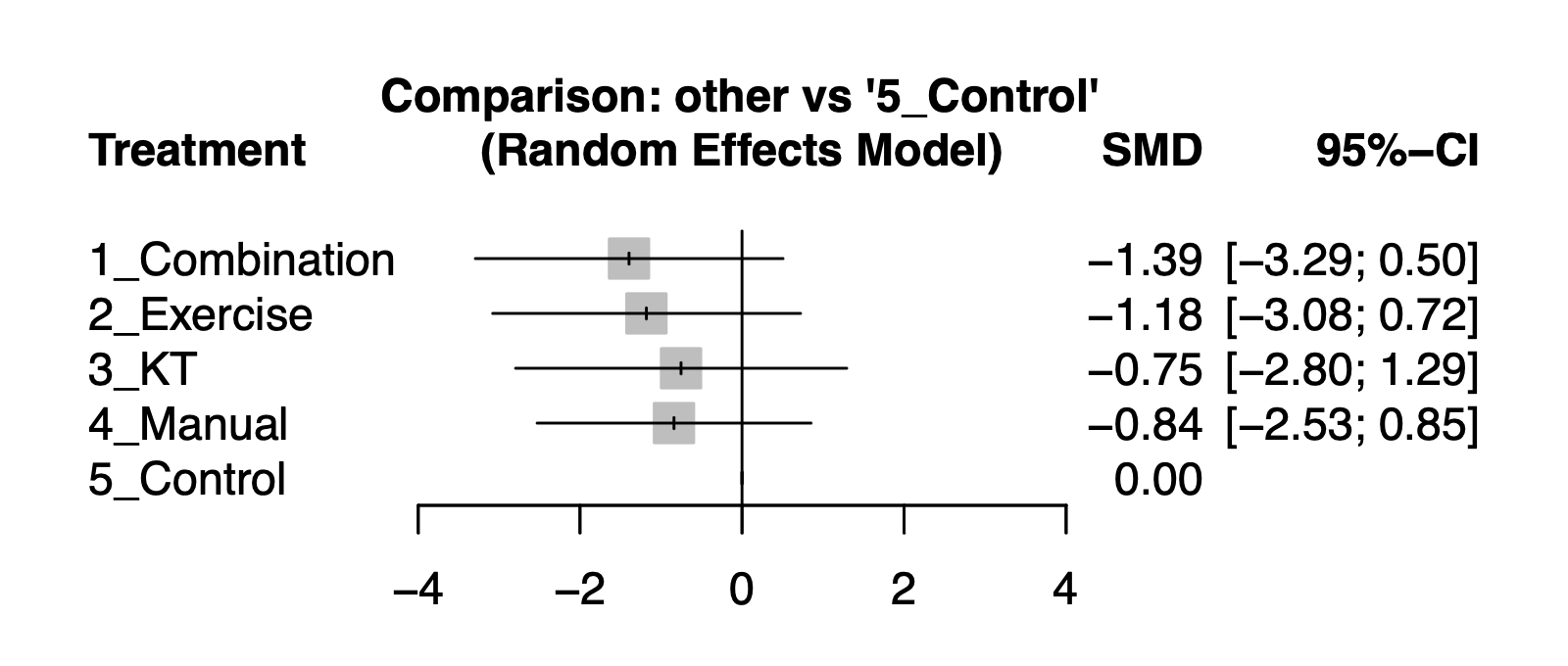

Supplement: Supplementary file 1 [file jcm-14-04765-s001.zip › FigS4d.tiff]

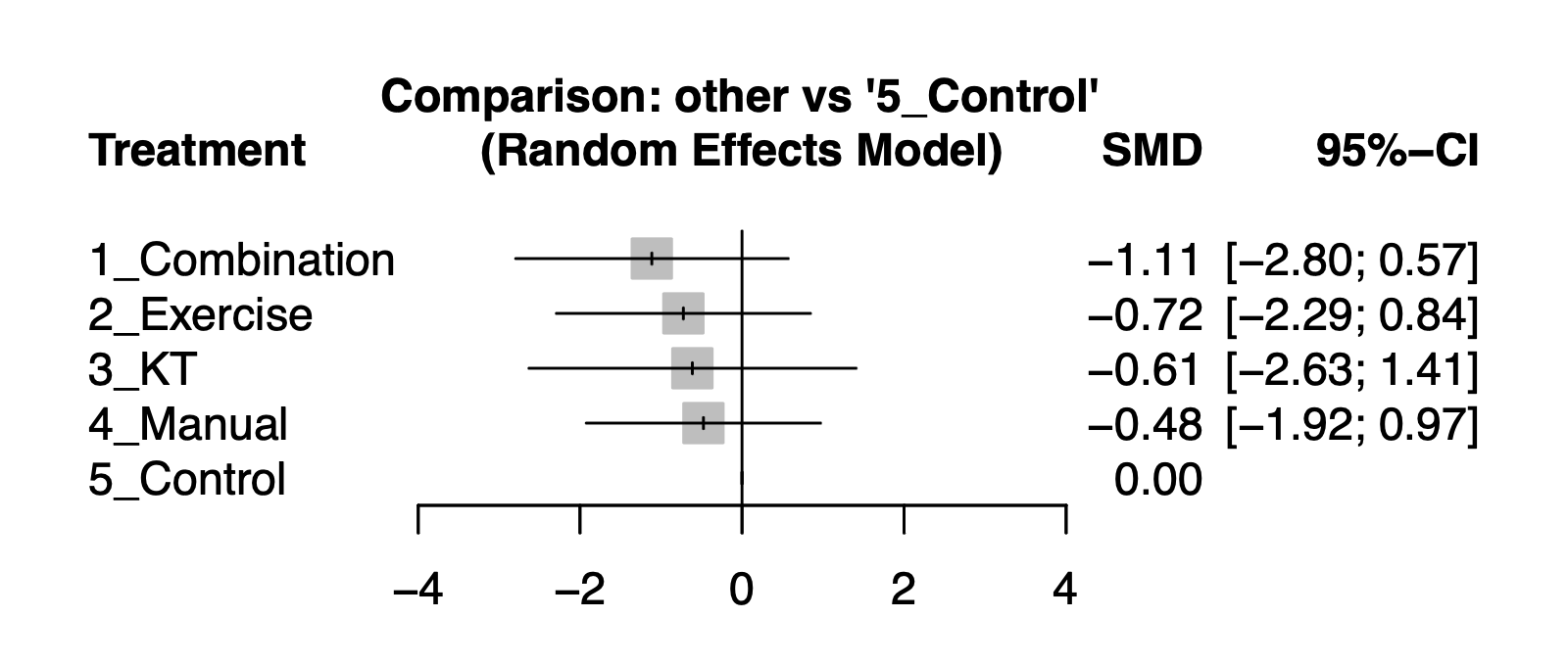

Supplement: Supplementary file 1 [file jcm-14-04765-s001.zip › FigS4e.tiff]

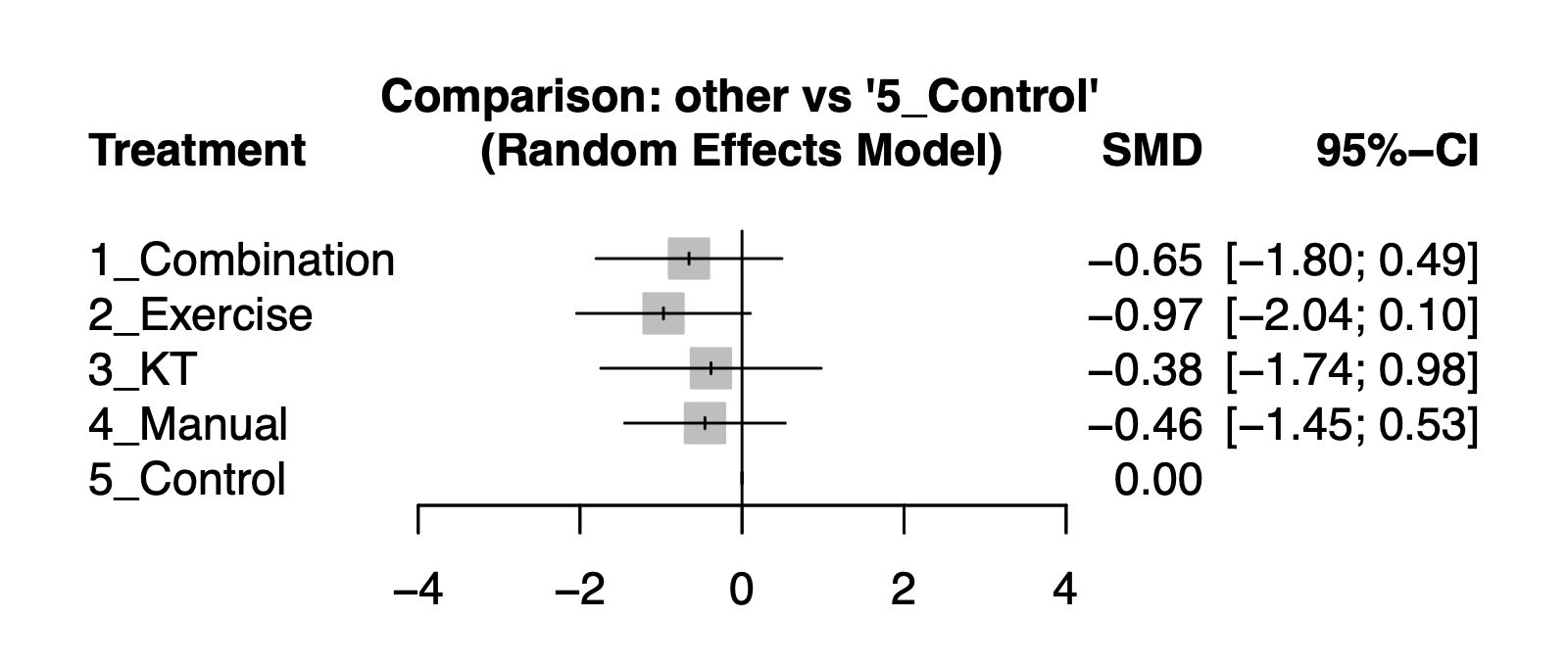

Supplement: Supplementary file 1 [file jcm-14-04765-s001.zip › FigS4f.tiff]

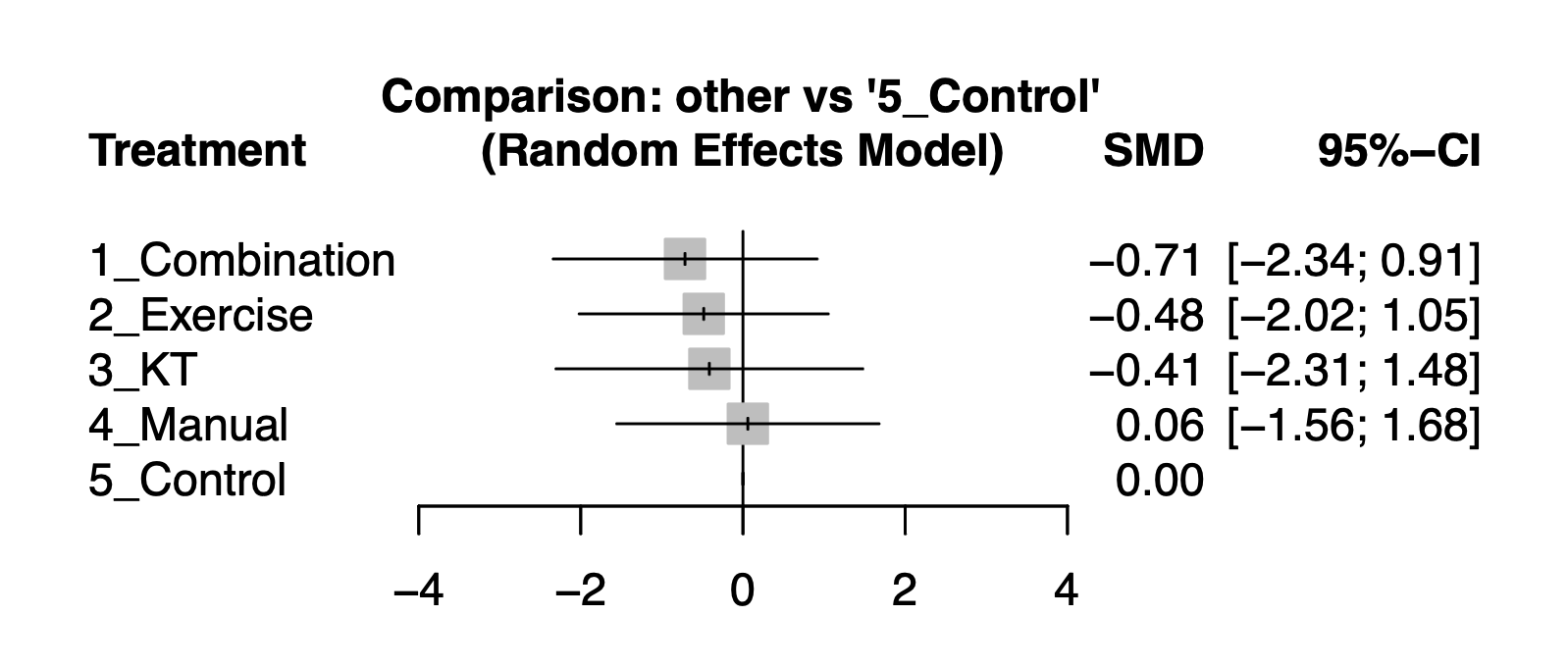

Supplement: Supplementary file 1 [file jcm-14-04765-s001.zip › FigS4g.tiff]

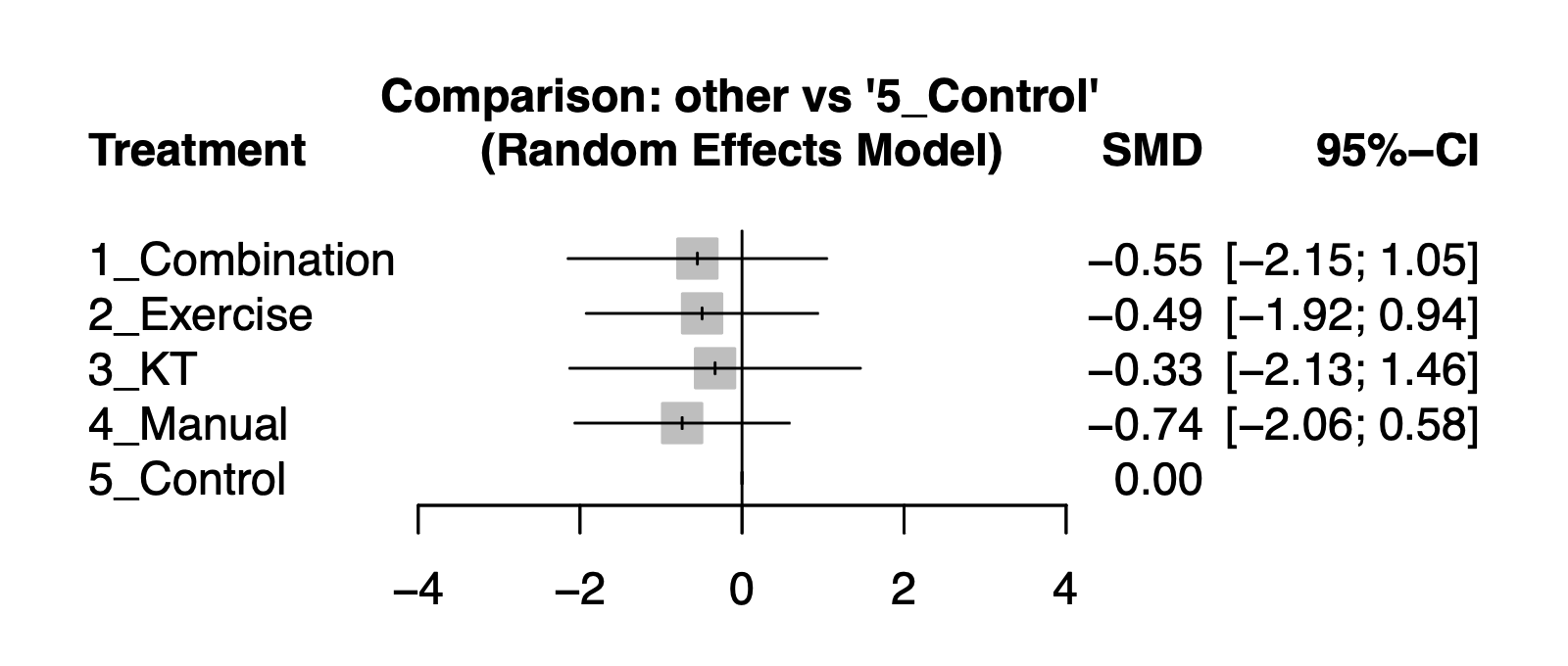

Supplement: Supplementary file 1 [file jcm-14-04765-s001.zip › FigS4h.tiff]

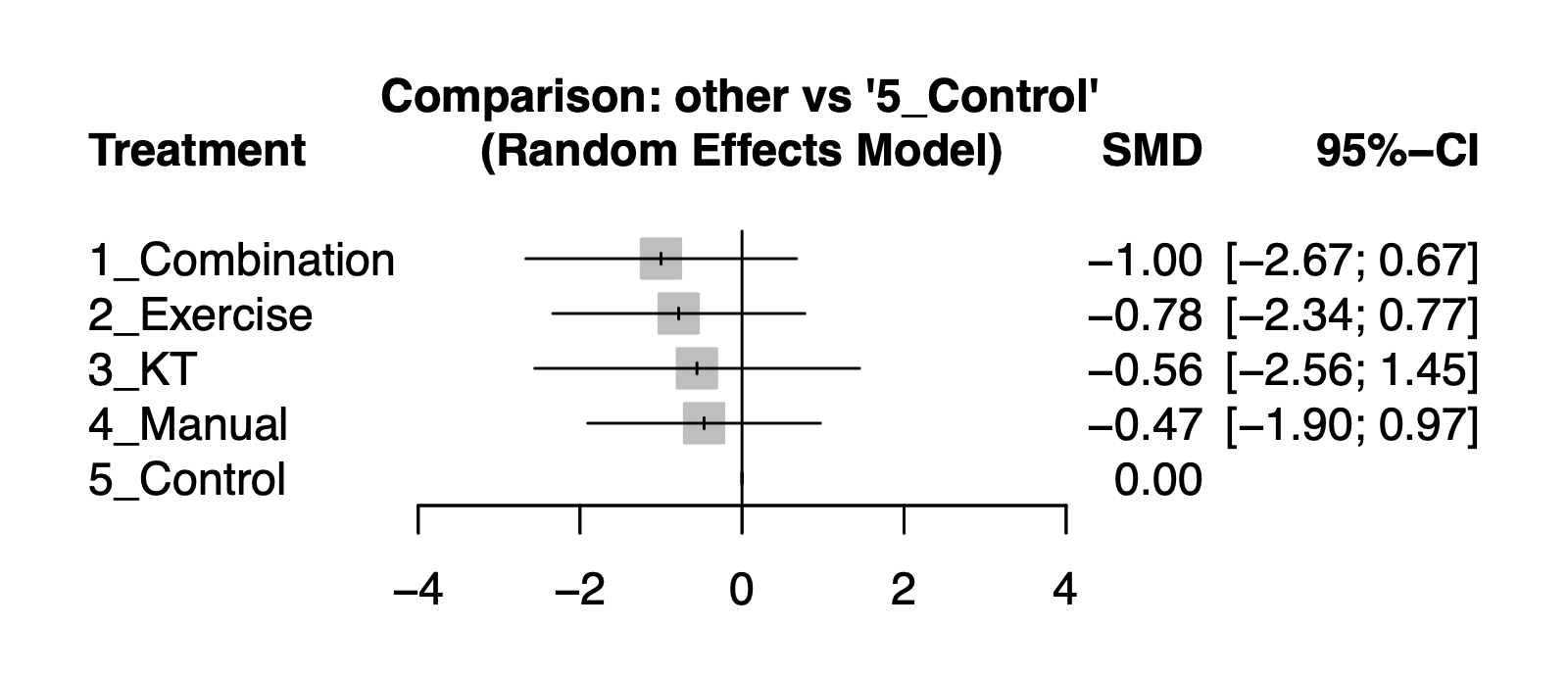

Supplement: Supplementary file 1 [file jcm-14-04765-s001.zip › FigS4i.tiff]

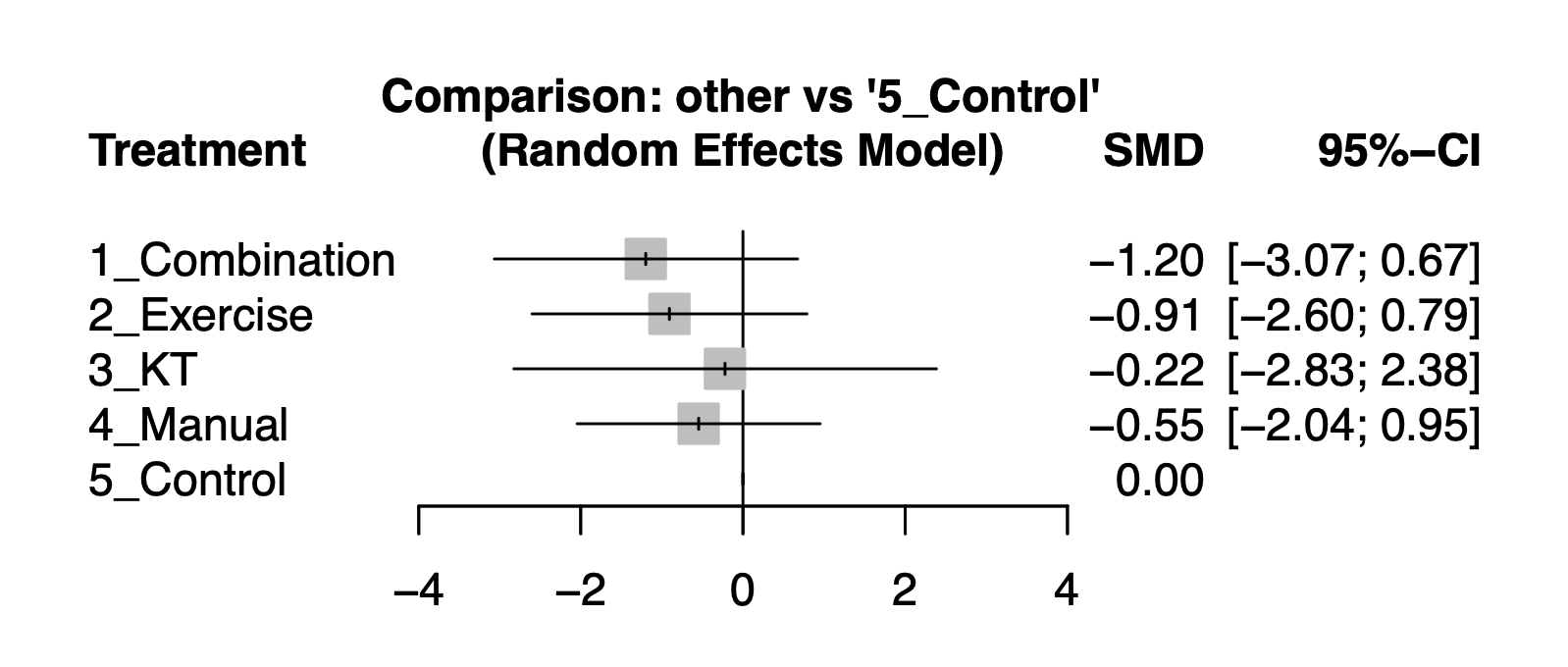

Supplement: Supplementary file 1 [file jcm-14-04765-s001.zip › FigS4j.tiff]

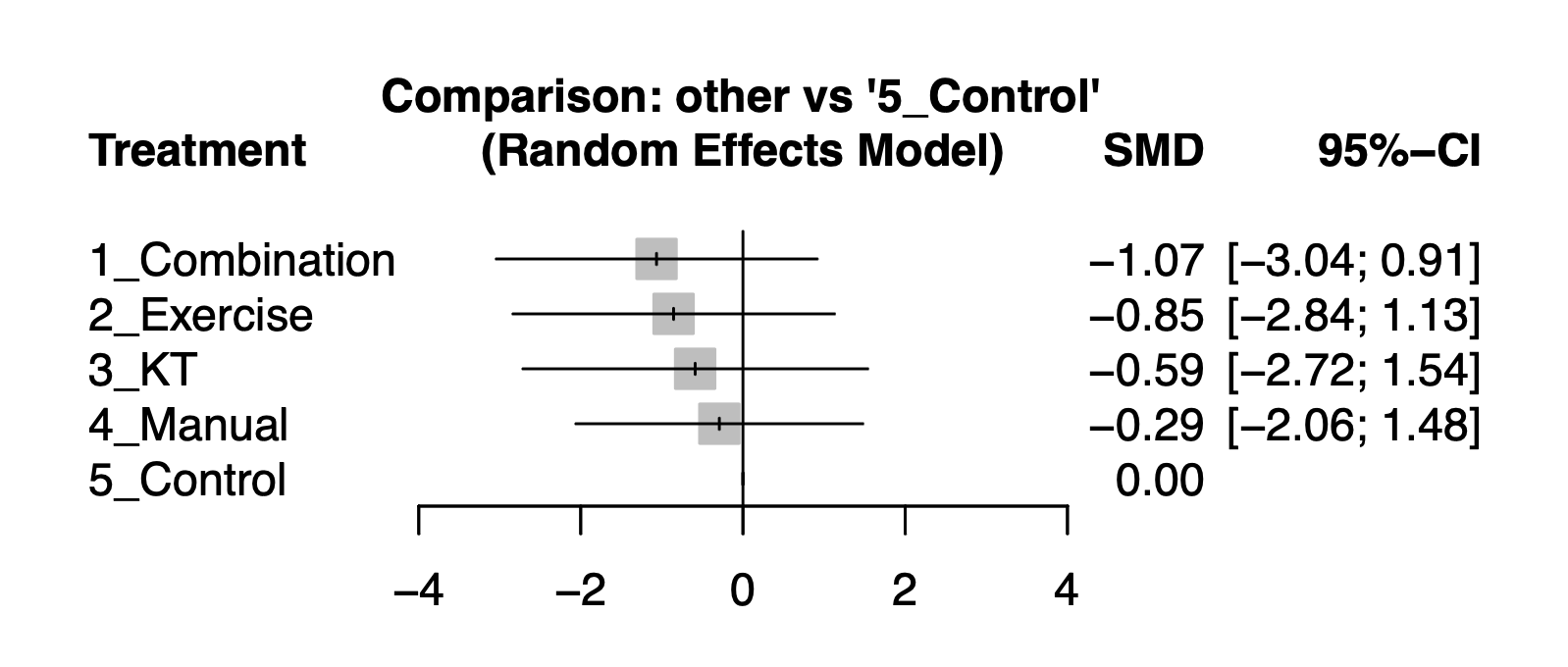

Supplement: Supplementary file 1 [file jcm-14-04765-s001.zip › FigS4k.tiff]

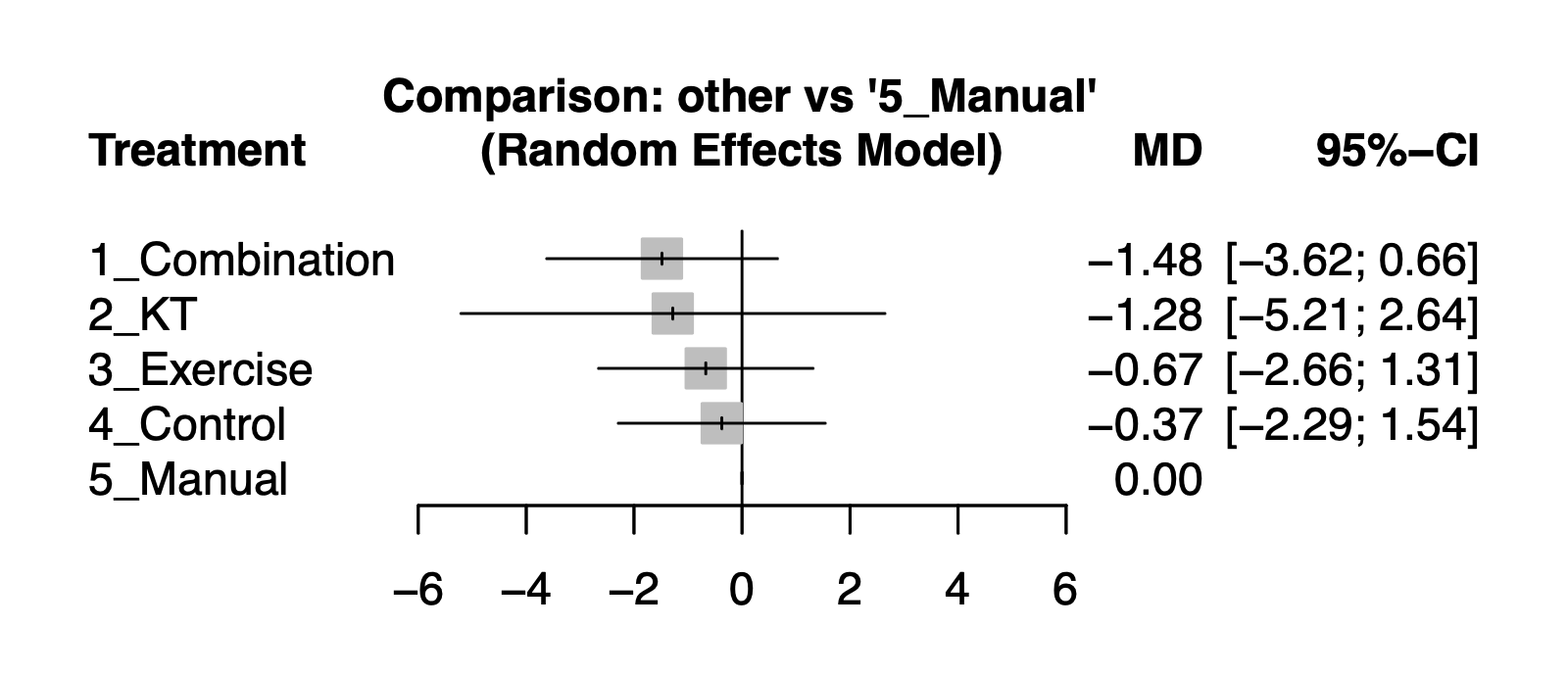

Supplement: Supplementary file 1 [file jcm-14-04765-s001.zip › FigS5a.tiff]

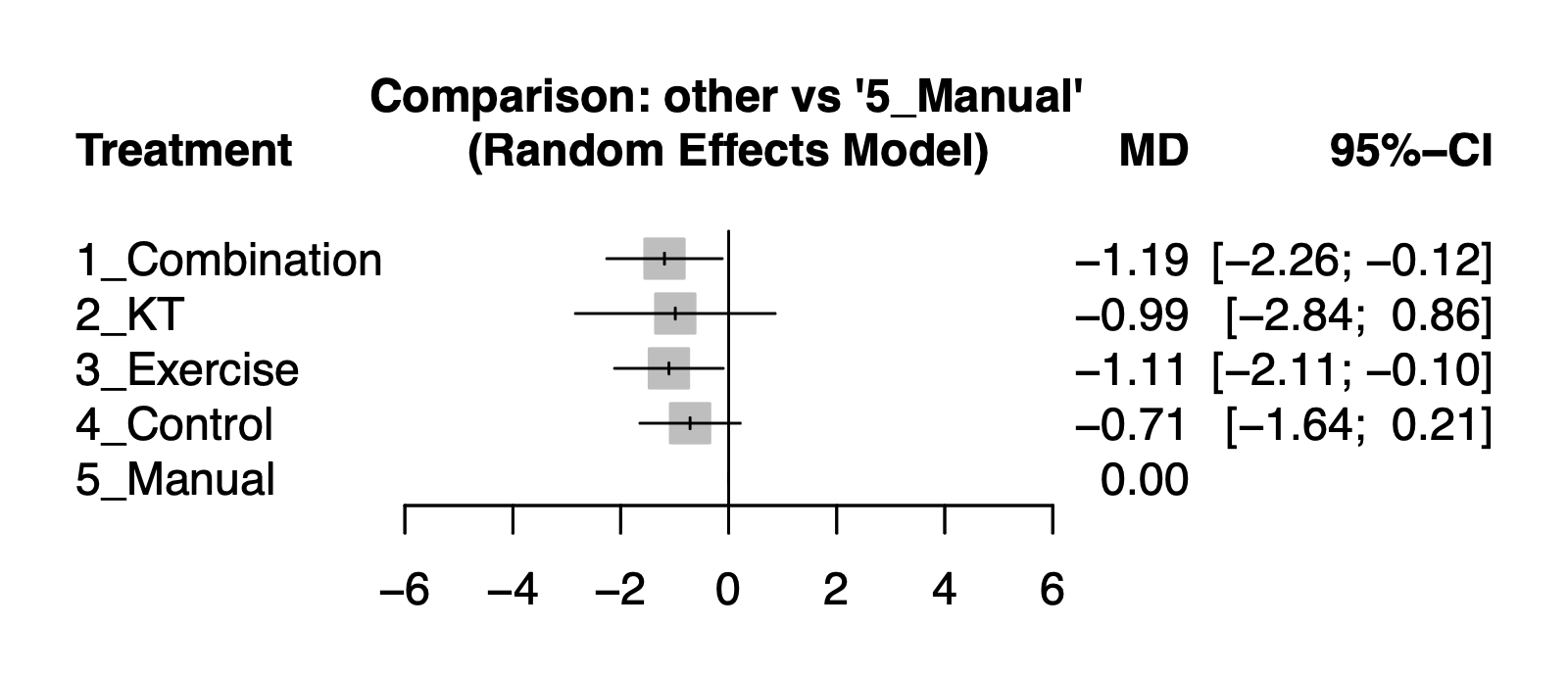

Supplement: Supplementary file 1 [file jcm-14-04765-s001.zip › FigS5b.tiff]

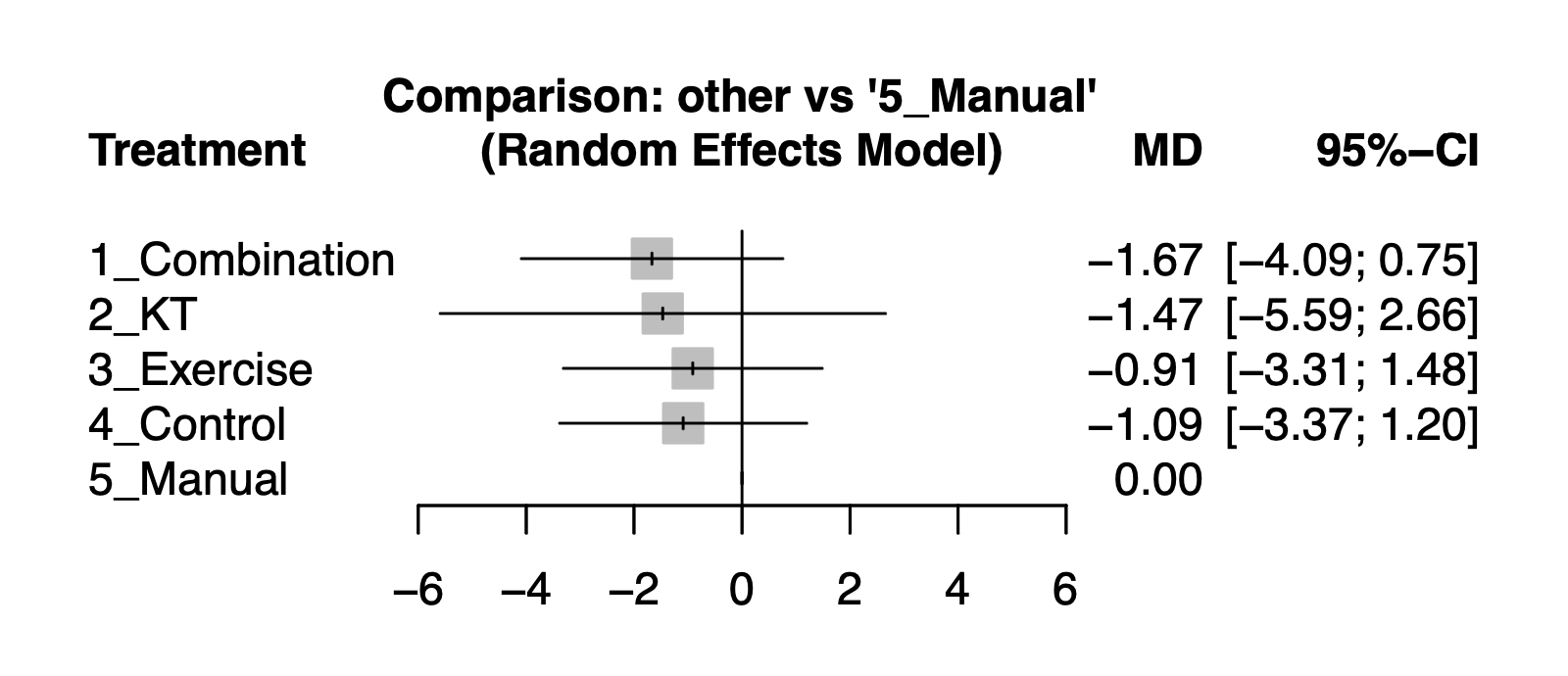

Supplement: Supplementary file 1 [file jcm-14-04765-s001.zip › FigS5c.tiff]

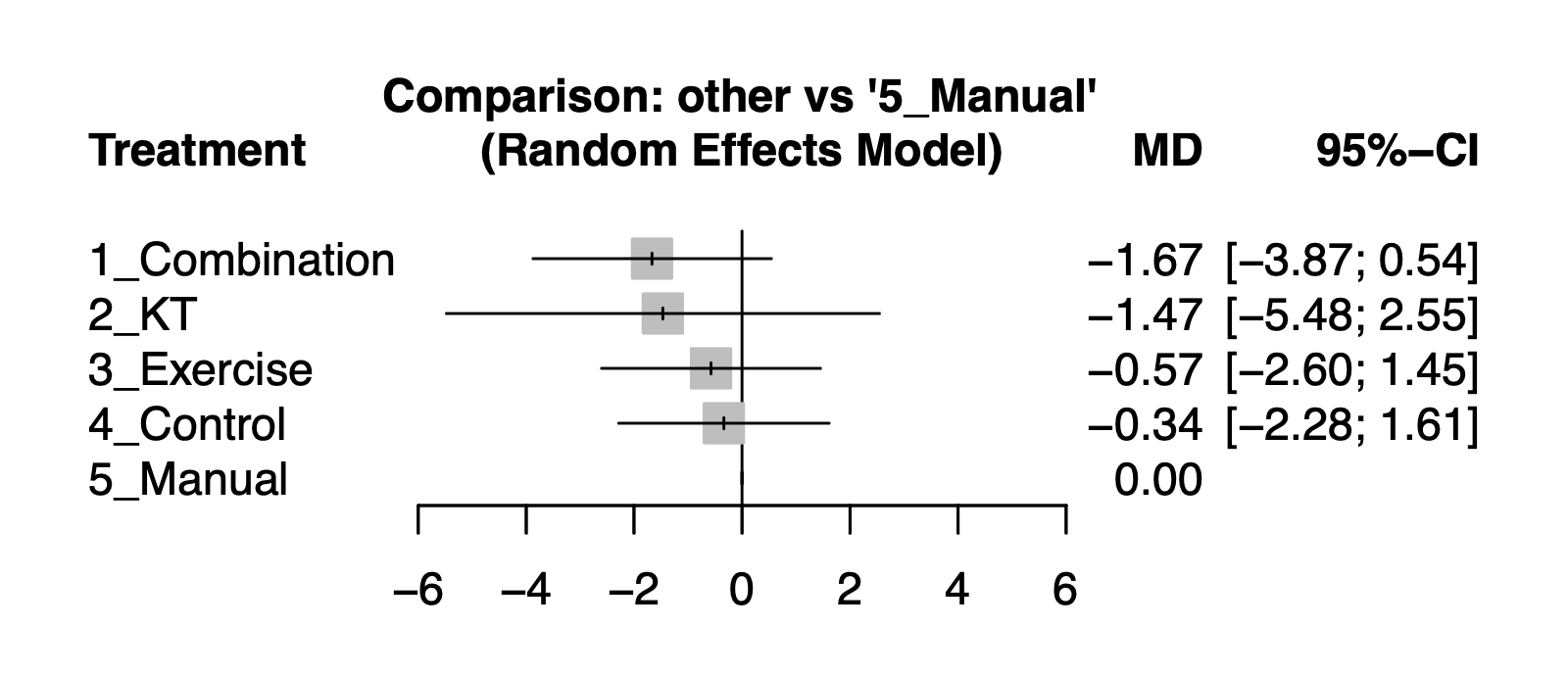

Supplement: Supplementary file 1 [file jcm-14-04765-s001.zip › FigS5d.tiff]

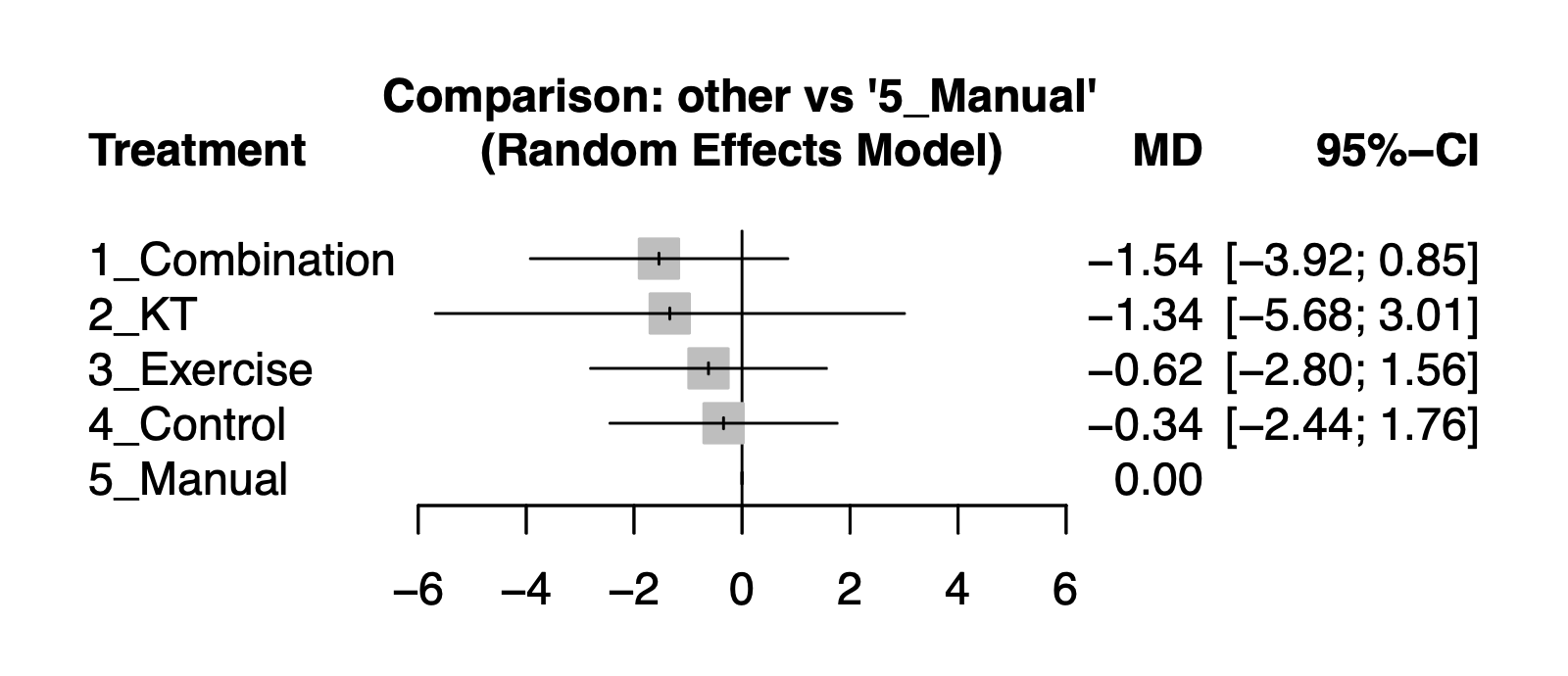

Supplement: Supplementary file 1 [file jcm-14-04765-s001.zip › FigS5e.tiff]

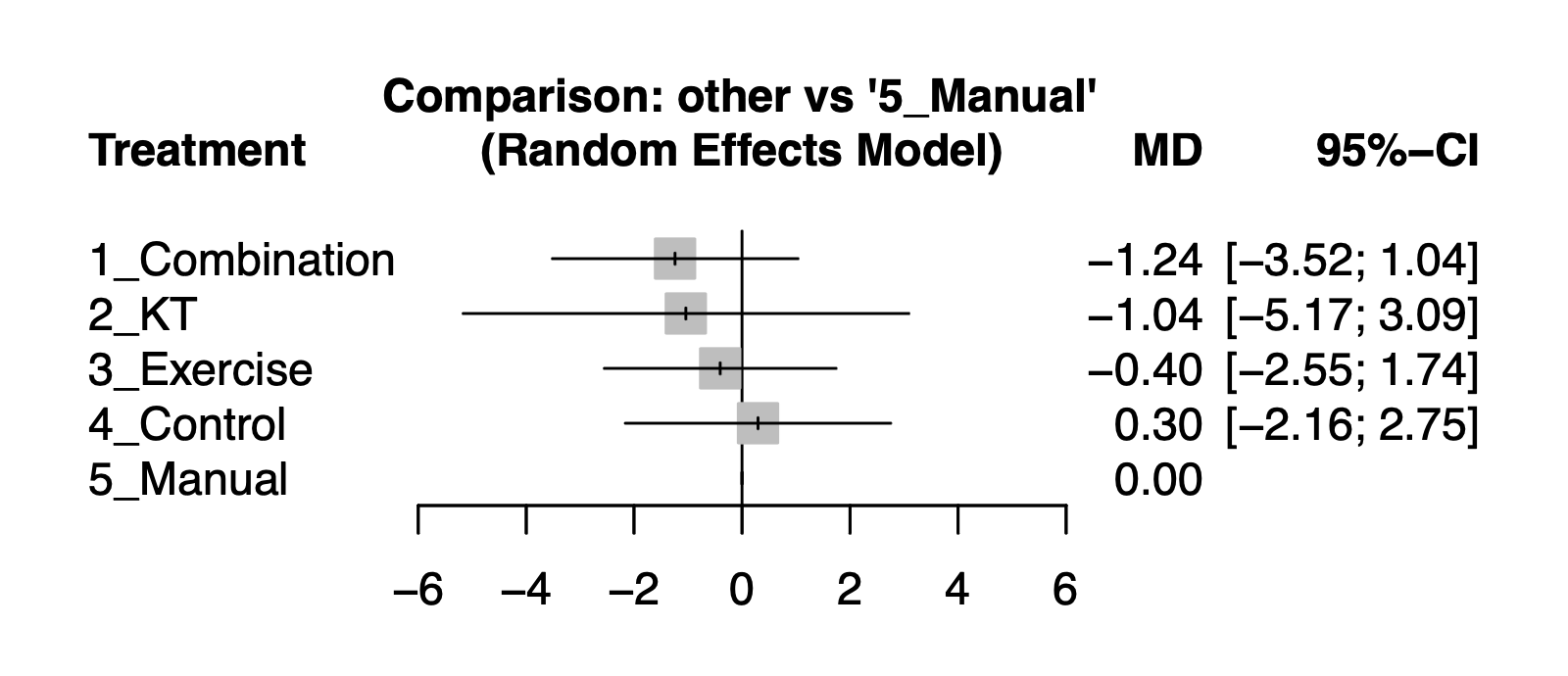

Supplement: Supplementary file 1 [file jcm-14-04765-s001.zip › FigS5f.tiff]

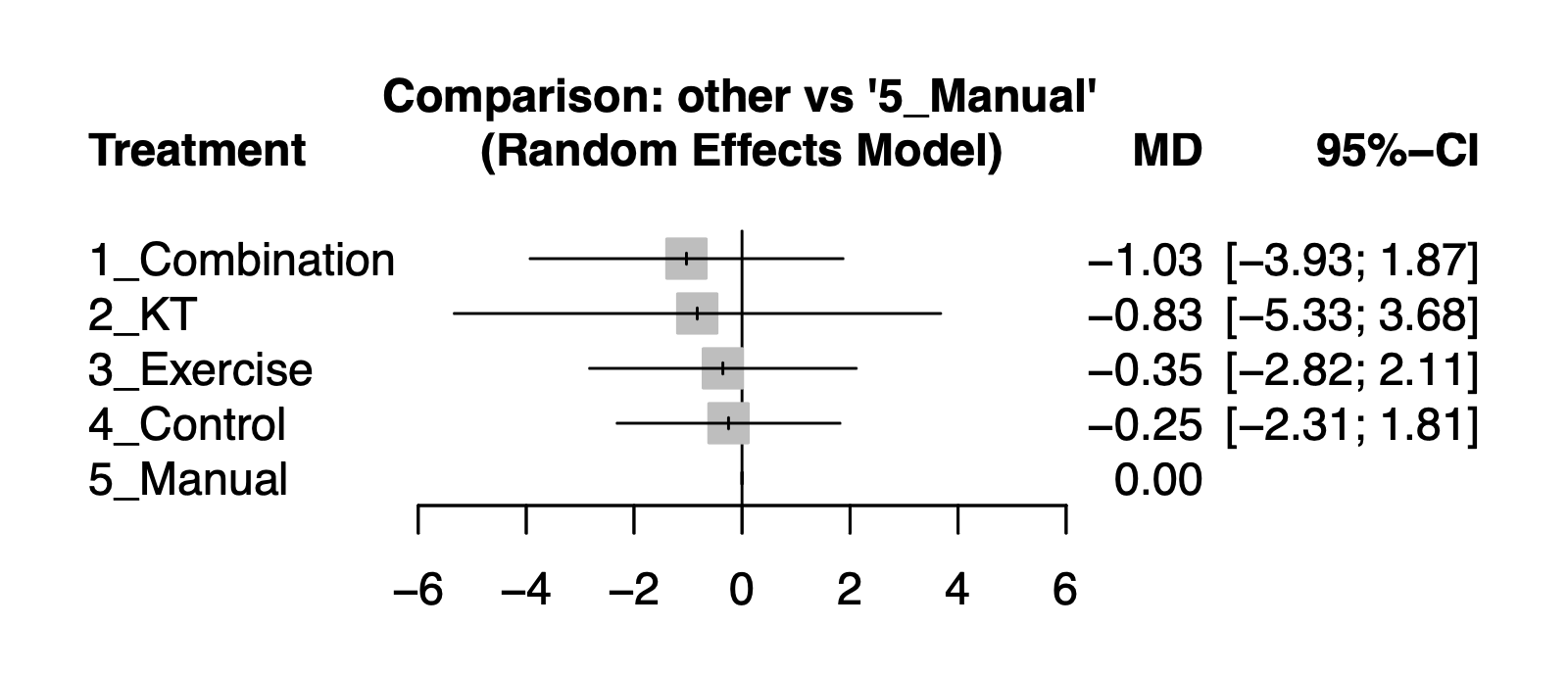

Supplement: Supplementary file 1 [file jcm-14-04765-s001.zip › FigS5g.tiff]

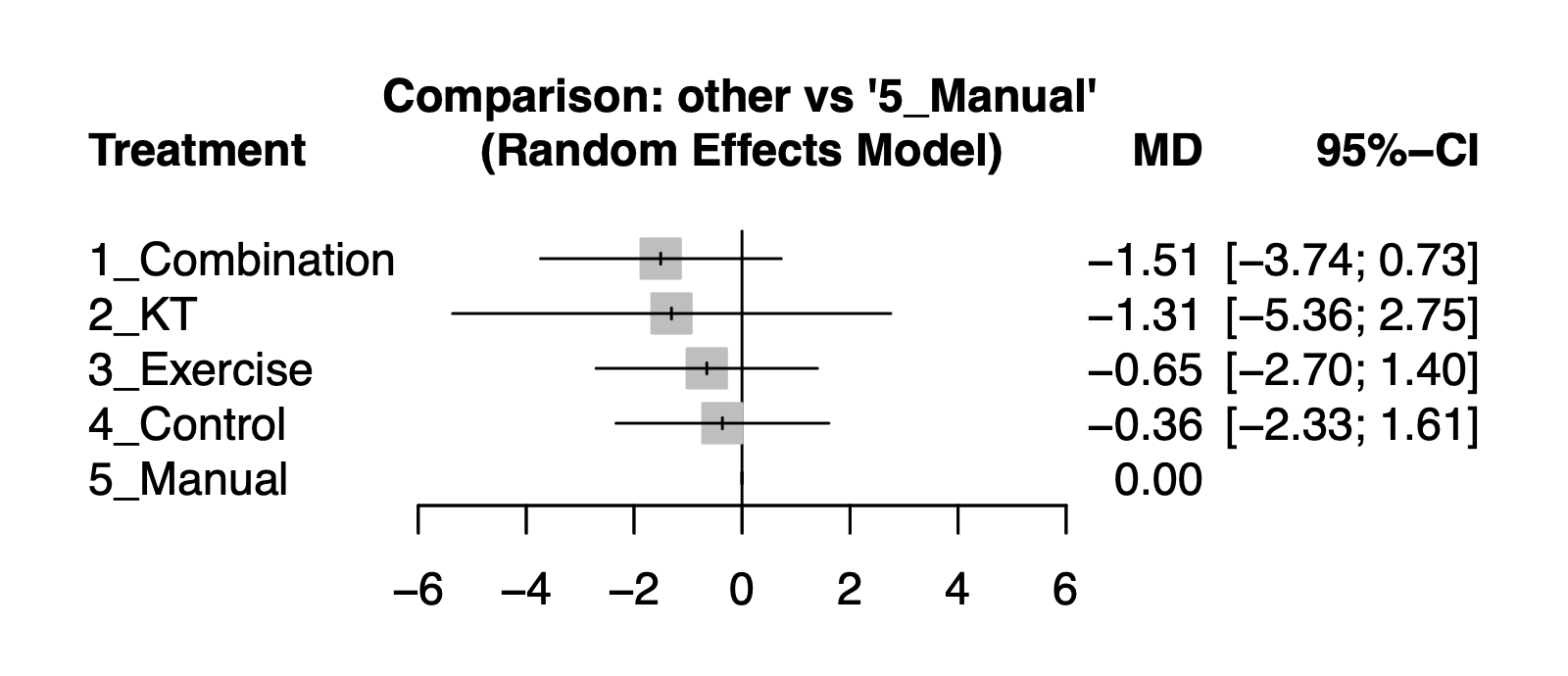

Supplement: Supplementary file 1 [file jcm-14-04765-s001.zip › FigS5h.tiff]

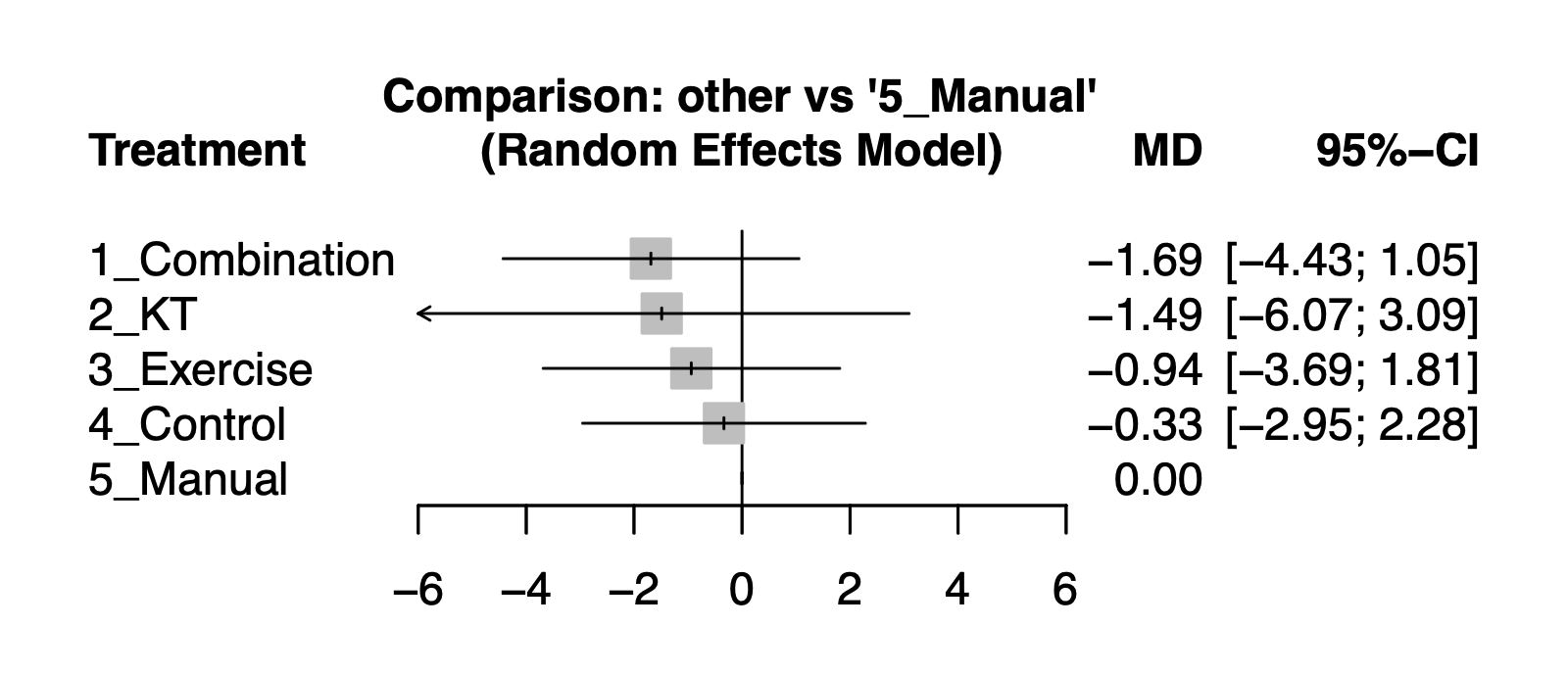

Supplement: Supplementary file 1 [file jcm-14-04765-s001.zip › FigS5i.tiff]

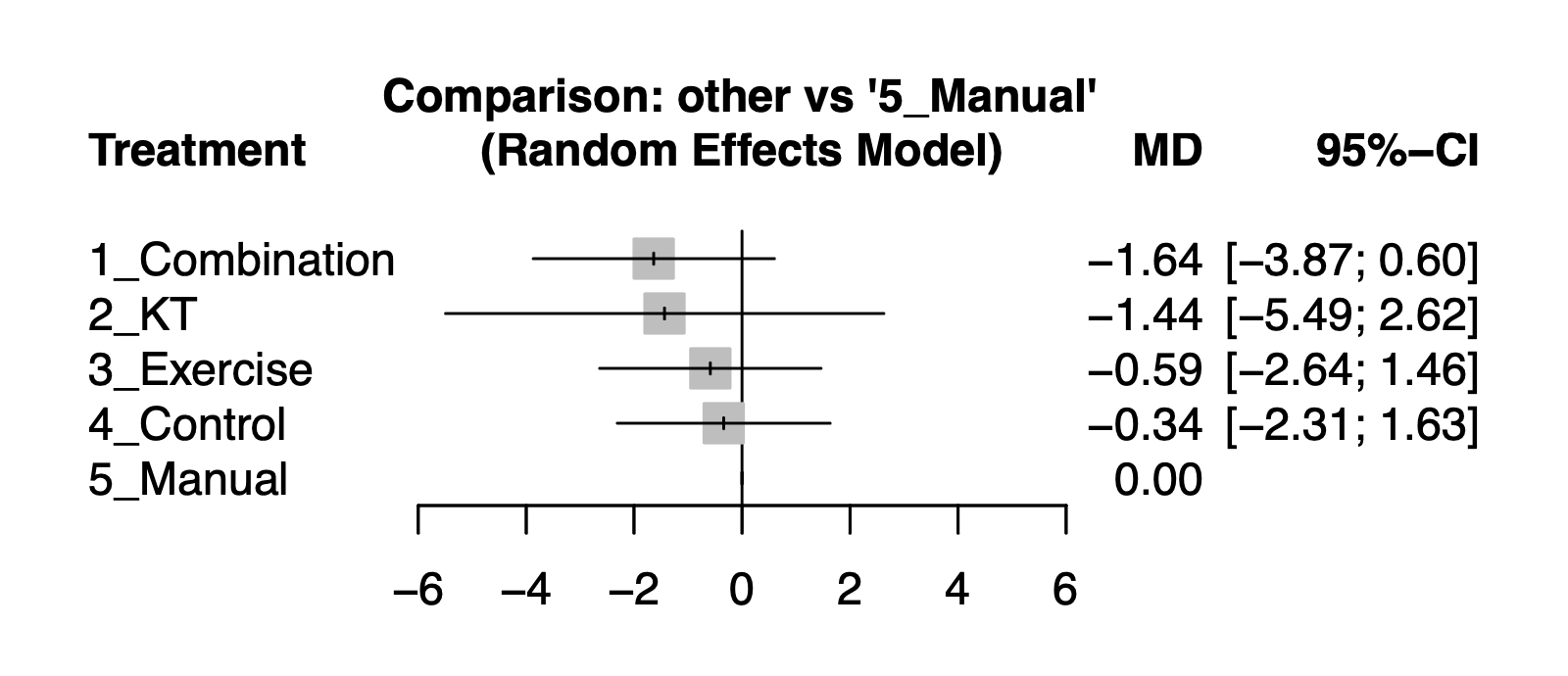

Supplement: Supplementary file 1 [file jcm-14-04765-s001.zip › FigS5j.tiff]
